# GOUHFI: A novel contrast- and resolution-agnostic segmentation tool for ultra-high-field MRI

**DOI:** 10.1162/IMAG.a.960

**Published:** 2025-10-24

**Authors:** Marc-Antoine Fortin, Anne Louise Kristoffersen, Michael Staff Larsen, Laurent Lamalle, Rüdiger Stirnberg, Pål Erik Goa

**Affiliations:** Department of Physics, Norwegian University of Science and Technology, Trondheim, Norway; Department of Computer Science, Norwegian University of Science and Technology, Trondheim, Norway; GIGA-CRC-Human Imaging, University of Liège, Liège, Belgium; German Center for Neurodegenerative Diseases (DZNE), Bonn, Germany; Department of Radiology and Nuclear Medicine, St. Olavs Hospital HF, Trondheim, Norway

**Keywords:** UHF-MRI, neuroimaging, brain segmentation, deep learning, contrast and resolution agnosticity, domain randomization

## Abstract

Recently, ultra-high-field MRI (UHF-MRI) has become more available and one of the best tools to study the brain for neuroscientists. One common step in quantitative neuroimaging is to segment the brain into several regions, which has been done using software packages such as *FreeSurfer*, *FastSurferVINN,* or SynthSeg. However, the differences between UHF-MRI and 1.5T or 3T images are such that the automatic segmentation techniques optimized at these field strengths usually produce unsatisfactory segmentation results for UHF images. Thus, it has been particularly challenging to perform region-based quantitative analyses as typically done with 1.5–3T data, considerably limiting the potential of UHF-MRI until now. Ultimately, this underscores the crucial need for developing new automatic segmentation techniques designed to handle UHF images. Hence, we propose a novel Deep Learning (DL)-based segmentation technique called GOUHFI: Generalized and Optimized segmentation tool for ultra-high-field images, designed to segment UHF images of various contrasts and resolutions. For training, we used a total of 206 label maps from four datasets acquired at 3T, 7T, and 9.4T. In contrast to most DL strategies, we used a previously proposed domain randomization approach, where synthetic images generated from the 206 label maps were used for training a 3D U-Net. This approach enables the DL model to become contrast agnostic. GOUHFI was tested on seven different datasets and compared with existing techniques such as *FastSurferVINN*, SynthSeg, and *CEREBRUM-7T*. GOUHFI was able to segment the six contrasts and seven resolutions tested at 3T, 7T, and 9.4T. Average Dice-Sørensen Similarity Coefficient (DSC) scores of 0.90, 0.90, and 0.93 were computed against the ground truth segmentations at 3T, 7T, and 9.4T, respectively. These results demonstrated GOUHFI’s superior performance to competing approaches at each resolution and contrast level tested. Moreover, GOUHFI demonstrated impressive resistance to the typical inhomogeneities observed at UHF-MRI, making it a new powerful segmentation tool allowing the usual quantitative analysis pipelines performed at lower fields to be applied also at UHF. Ultimately, GOUHFI is a promising new segmentation tool, being the first of its kind proposing a contrast- and resolution-agnostic alternative for UHF-MRI without requiring fine tuning or retraining, making it the forthcoming alternative for neuroscientists working with UHF-MRI or even lower field strengths.

## 1 Introduction

One of the most important steps in quantitative neuroimaging pipelines is the segmentation of the brain into its different regions. Segmentation can be used to identify specific brain regions for cognitive disease diagnosis, to perform quantitative analyses like relaxometry or volumetry, and to help with surgical planning or image-guided interventions (Despotović et al., 2015; González-Villà et al., 2016; Klinger et al., 2024; Singh & Singh, 2021). Due to the considerable amount of time and expertise required to produce manual segmentations for many regions and subjects, automatic methods have been developed. Historically, atlas- and Bayesian-based techniques have been proposed, such as MABMIS and *FreeSurfer* or FSL-FIRST, respectively (Fischl et al., 2002; Jia et al., 2012; Patenaude et al., 2011). All propose to automatically segment the whole brain into several cortical and subcortical labels. However, with the developments in graphical processing units (GPU) in the last decade, Deep Learning (DL) has drastically changed the landscape of automatic brain segmentation. Whereas regular machine learning (ML) approaches have shown limited ability to generalize and adapt to complex imaging modalities, convolutional neural networks (CNN) used for DL models have become increasingly successful in handling these challenges (Singh & Singh, 2021). More precisely, the U-Net architecture proposed by Ronneberger et al. (2015) has shown remarkable performance for brain segmentation tasks. Due to its symmetrical encoder–decoder structure with skip connections, creating a U-shaped architecture, the U-Net is able to efficiently extract features at different scales in the images. Recently, several techniques using the U-Net architecture have been proposed such as AssemblyNet (Coupé et al., 2020), QuickNat (A. G. Roy et al., 2019), SLANT27 (Huo et al., 2019), FastSurferCNN (Henschel et al., 2020), and FastSurferVINN (Henschel et al., 2022), which allow the segmentation of the brain into more than 25 labels.

Most of the DL-based brain segmentation techniques, including all of the aforementioned ones, rely on the typical paradigm of using a T1w input image with its corresponding segmentation/label map as training data. In order to improve the network capacity to generalize to unseen T1w images and increase the training corpus size, extensive data augmentation (DA) is applied on the training data. However, generalization to unseen contrasts and resolutions has shown limitations where segmentation performance quickly decreases when used on images outside the training domain (Ghafoorian et al., 2017; Karani et al., 2018). This limitation is known as the “domain gap” problem (Pan & Yang, 2009). While this issue can be partially addressed by having multi-modality training data or test–time domain adaptation methods (Havaei et al., 2016; Karani et al., 2021), the network will still struggle when encountering completely unseen images. Alternatively, fine-tuning these models to new contrasts has shown great results. Ultimately, since this fine-tuning is required for every new contrast, this quickly becomes limiting in practice and not an “out-of-the-box” solution.

Historically, contrast invariance for brain MRI segmentation has been successfully achieved through Bayesian segmentation (Van Leemput et al., 1999). However, this approach requires considerably more computational time than DL-based techniques. The fastest Bayesian techniques can process one subject in ∼15 minutes (Puonti et al., 2016), whereas DL-based techniques require less than 1 minute. Consequently, contrast-invariant Bayesian segmentation techniques have been extremely challenging to implement in clinical settings.

Thus, a novel paradigm for DL training data where randomly generated synthetic images are used instead of real images has emerged. This approach is called domain randomization (DR) and was proposed for brain segmentation for the first time by Billot et al. (2023). More precisely, synthetic images are generated directly from label maps, using a fully randomized generative model creating images with random contrasts and augmentations that are far beyond what is actually realistic. In Billot et al. (2023), a novel segmentation tool, SynthSeg, was proposed where this DR approach was combined with a 3D U-Net in order to segment MR brain images. SynthSeg demonstrated remarkable generalization to unseen contrasts and images with low signal-to-noise ratio (SNR) without the need for fine-tuning or retraining. Moreover, SynthSeg outperformed the state-of-the-art Bayesian approach SAMSEG (Puonti et al., 2016) in all tested datasets, in addition to being substantially faster. As a result, the approach proposed by SynthSeg has recently been used for other applications such as segmentation of white matter (WM) lesions or neonatal brain (Gibson et al., 2024; Valabregue et al., 2024), and is widely available through the FreeSurfer package and distributed with MATLAB (from R2022b and onward).

While both paradigms (real images + DA vs. synthetic images + DR) have been used for many different applications, none of them have been applied to UHF-MRI (i.e., ≥7T). UHF-MRI accessibility has increased in the last decade and has even been used for large neuroimaging studies such as the Human Connectome Project (HCP) due to its higher SNR, contrast, and spatial resolution (Trattnig et al., 2018). Despite the several advantages of UHF-MRI, UHF images typically suffer from significant transmit radiofrequency (RF) inhomogeneities compared with lower field strengths, due to the shorter RF wavelength (Schick, 2005). This results in significant signal and contrast inhomogeneities observed across the image (Webb & Collins, 2010). Although recent developments in parallel transmit (pTx) RF pulses have substantially improved both signal and contrast homogeneity compared with single transmit (1Tx) pulses (Gras et al., 2017), pTx pulses are not widely available and have yet to be applied in large neuroimaging studies.

This inaccessibility to large datasets with homogeneous UHF images has considerably hindered the development of typical DL-based techniques from T1w images. Only one technique, CEREBRUM-7T, has been especially designed to segment 7T T1w MP2RAGE images (Svanera et al., 2021). Without retraining or fine-tuning, CEREBRUM-7T can segment (0.63 mm)^3^ T1w MP2RAGE from the Glasgow dataset with a matrix shape of 256 × 352 × 224 into six labels: white matter (WM), gray matter (GM), ventricles, basal ganglia, cerebellum, and brainstem. Alternatively, considering the limited access to UHF-designed segmentation techniques, several studies have been compelled to use 3T-designed tools such as FreeSurfer on 7T data by implementing extensive preprocessing on the images (Zaretskaya et al., 2018). Additionally, FastSurferVINN, which proposes a solution for sub-millimeter T1w images at 3T, has also been recently tested at 7T with pTx T1w images and has shown promising results (Cabalo et al., 2025; Fortin et al., 2025). Ultimately, while tools designed at 3T can be a solution for specific UHF T1w images acquired with pTx, they do not provide a reliable solution for most UHF data. Indeed, when both signal and contrast inhomogeneities and resolution differences with 3T are combined, segmentation results are frequently unsatisfactory, requiring important visual quality assurance (QA) and even extremely time-consuming manual corrections.

Thus, considering the recent increased accessibility of UHF-MRI, there is an urgent need for developing novel automatic segmentation techniques able to address the new issues introduced with UHF-MRI. To the best of our knowledge, no DL technique currently exists to segment (1) T1w UHF images in more than six labels, (2) highly inhomogeneous 1Tx UHF images, or (3) non-T1w contrast UHF images.

In this work, we propose GOUHFI: Generalized and Optimized segmentation tool for ultra-high-field images. By adapting the DR approach proposed in Billot et al. (2023) to the UHF-MRI context and using a state-of-the-art DL architecture with an extensive training corpus, GOUHFI is able to segment UHF images of various contrasts and resolutions in clinically feasible times without fine-tuning or retraining. More precisely, we present in detail how GOUHFI was developed and trained, in addition to present its in-depth quantitative and qualitative evaluation against two other segmentation techniques at 3T and 7T. Furthermore, GOUHFI’s performance against manual delineations at 9.4T and clinical relevance in volumetry measurements between Parkinson’s disease patients and healthy controls was evaluated.

## 2 Methods

### 2.1 Datasets

After conducting a comprehensive review of all sub-millimeter MRI datasets freely available online, the eight following datasets were selected for training and testing GOUHFI. An overview of all these datasets is available in Table 1.

**Table 1.** Summary of the datasets used for training and/or testing in this work.

Dataset	Field Strength	Resolution	Contrast	Subjects	Vendor	Use	N
HCP-YA	3T	(0.7 mm)^3^	T1w/T2w	Healthy	Siemens	Tr	80/20
SCAIFIELD	7T (pTx)	(0.6 mm)^3^	T1w, MPM-T1w,-MTw,-PDw	Healthy	Siemens	Tr/Ts	31/10
UltraCortex	9.4T (1Tx)	(0.6 mm)^3^/(0.8 mm)^3^	T1w	Healthy	Siemens	Tr/Ts	15/12
ABIDE-II ETHZ	3T	(0.9 mm)^3^	T1w	ASD	Philips	Tr	34
ABIDE-II EMC	3T	(0.9 mm)^3^	T1w	ASD	GE	Tr	46
MPI-CBS	7T (1Tx)	(0.4 mm)^3^	T1w	Healthy	Siemens	Ts	28
STRAT-PARK	7T (1Tx)	(0.75 mm)^3^	T1w	PDP/Healthy	Siemens	Ts	45
CEREBRUM-7T	7T (1Tx)	(0.63 mm)^3^	T1w	Healthy	Siemens	Ts	21
Human Brain Atlas	7T (1Tx)	(0.25 mm)^3^	T1w	Healthy	Siemens	Ts	1

The table lists the field strength, resolution, contrast, subject type, vendor, usage, and number of subjects for each dataset. ASD: autism spectrum disorder, PDP: Parkinson’s disease patients, Tr: training, Ts: test.

#### 2.1.1 Human Connectome Project: Young Adult

The Human Connectome Project Young Adult (HCP-YA) (Van Essen et al., 2012) is a large neuroimaging study including structural and functional MR images obtained at 3T and 7T on healthy participants between the ages of 22 and 35 years. For GOUHFI, a subset of 100 randomly selected subjects with preprocessed structural (0.7 mm)^3^ T1w MPRAGE and T2w SPACE images acquired at 3T were used. The preprocessing steps included gradient distortion correction, coregistration, and averaging of both T1w and T2w runs individually (each sequence is acquired twice per session), Anterior Commissure-Posterior Commissure (ACPC) registration, brain extraction, field map distortion correction, coregistration of T2w to the T1w, and a bias field correction. More details on the acquisition parameters of both MPRAGE and SPACE sequences and preprocessing steps can be found online.^*^ The complete dataset is freely available online.^†^ In this work, 80 subjects were used for training and 20 for testing.

#### 2.1.2 SpinoCerebellar Ataxias: Advanced Imaging with ultra-high-FIELD MRI

The SpinoCerebellar Ataxias: advanced imaging with ultra-high-FIELD MRI (SCAIFIELD) is a project aiming at establishing quantitative UHF-MRI biomarkers for polyglutamine SCAs.^‡^ For this purpose, a multi-center study has been conducted on 41 healthy participants with data acquired on two 7T MAGNETOM Terra and one 7TPlus MAGNETOM scanners (Siemens Healthineers, Erlangen, Germany) with the same 8Tx/32Rx head coil model in pTx mode. All sequences were acquired using Universal pTx RF Pulses (UP) (Gras et al., 2017) created from a database of B_0_ and ${\text{B}}_{1}^{+}$ maps acquired at each partner site. The imaging protocol included acquisition of a Multi-Parameter-Mapping (MPM) dataset consisting of Magnetization Transfer-, T1- and Proton Density-weighted multi-echo spoiled gradient echo contrasts, and a T1w MPRAGE, all at (0.6 mm)^3^ resolution. For this study, 31 subjects were used for training and 10 for testing. The first echo time images of the MPM images were also used for testing (denoted MPM-MTw, MPM-T1w, and MPM-PDw).

#### 2.1.3 UltraCortex

The UltraCortex (Mahler et al., 2025) is a collaborative project between the Max Planck Institute for biological Cybernetics’ High-Field Magnetic Resonance and University Hospital Tübingen’s Biomedical Magnetic Resonance Departments providing MR images acquired at 9.4T on 78 healthy adult volunteers (M/F: 50/28, age range: 20–53 years old). In total, 86 examinations were performed with either the MPRAGE (n = 18) or MP2RAGE (n = 68) sequence with sub-millimeter resolutions of (0.6 mm)^3^, (0.7 mm)^3^, and (0.8 mm)^3^, depending on the subject.^§^ The images were acquired on a 9.4T whole-body MRI scanner (Siemens Healthineers, Erlangen, Germany) with a 16-channel dual-row transmit array operating in CP+ mode paired with a 31-channel receive array. For MP2RAGE, the images were ${\text{B}}_{1}^{+}$ corrected and the background noise was removed using the regularization approach proposed in O’Brien et al. (2014). All images were skull stripped using *SynthStrip* (Hoopes et al., 2022). Additionally, a set of manual segmentations for WM and GM is provided for 12 subjects which was used as a test dataset for this study (n = 8 (0.6 mm)^3^ MP2RAGE, n = 1 (0.8 mm)^3^ MP2RAGE and n = 3 (0.6 mm)^3^ MPRAGE). These manual labels were first produced by FreeSurfer, manually corrected by student assistants and then validated by two expert neuroradiologists. More details on the data acquisition and processing can be accessed in Mahler et al. (2025).

#### 2.1.4 Autism Brain Imaging Data Exchange (ABIDE) II

The Autism Brain Imaging Data Exchange (ABIDE) II (Di Martino et al., 2017) is a large 3T dataset containing 1114 subjects across 19 institutions with different autism spectrum disorders freely available online^**^. In this work, two sub-cohorts using T1w images at (0.9 mm)^3^ resolutions were used. The first one, named ETHZ, included 34 subjects acquired with a 3T Philips Achieva scanner (Philips Healthcare, Best, Netherlands) at ETH Zurich. The second sub-cohort, EMC, acquired 46 subjects with a 3T GE MRI scanner (General Electric Discovery MR750, Milwaukee, MI, USA) at the Erasmus University Medical Center in Rotterdam. More details about the scanning procedure and parameters can be obtained by following the link provided above. All images from both sub-cohorts were used for training.

#### 2.1.5 Max Planck Institute for Human Cognitive Brain Sciences

The Open Science CBS Neuroimaging Repository is a dataset repository containing high-resolution and quantitative MRI data acquired at 7T, with single-transmit channel, at the Max Planck Institute for Human Cognitive Brain Sciences (MPI-CBS) in Leipzig (Tardif et al., 2016). The dataset includes 28 MP2RAGE images acquired on healthy subjects (M/F: 13/15, age: 26 ± 4 years old) at (0.5 mm)^3^ but reconstructed at a resolution of (0.4 mm)^3^. All shared images have previously been skull stripped.

#### 2.1.6 STRAT-PARK

The START-PARK cohort (Stige et al., 2024) is a large ongoing initiative trying to stratify Parkinson’s disease (PD) using a multi-disciplinary and multi-center longitudinal cohort composed of PD and neurologically healthy control individuals from Norway and Canada. One branch of STRAT-PARK proposes to use 7T MRI to stratify PD individuals using a high-resolution, multi-contrast, and quantitative protocol including both anatomical and functional images. As part of the imaging protocol, a (0.75 mm)^3^ MP2RAGE was acquired with a 7T MAGNETOM Terra scanner (Siemens Healthineers, Erlangen, Germany) using 1Tx channel. The MP2RAGE has been skull stripped in previous work done locally. For this project, a total of 45 subjects were used for testing with 24 PD patients (PDP) (M/F: 13/11, age: 66 ± 7 years old) and 21 healthy controls (HC) (M/F: 10/11, age: 60 ± 9 years old).

#### 2.1.7 CEREBRUM-7T: Glasgow dataset

As part of the work presented in Svanera et al. (2021), the test dataset used to assess CEREBRUM-7T’s performance composed of 21 scanning sessions (11 subjects) acquired with a Siemens 7T Terra MAGNETOM scanner at the Queen Elizabeth University Hospital (Glasgow, UK) was made available online.^††^ Each session contains a (0.63 mm)^3^ 1Tx T1w MP2RAGE and the automatic segmentations computed by CEREBRUM-7T. All 21 examinations were used for testing GOUHFI against CEREBRUM-7T.

#### 2.1.8 Human Brain Atlas

The Human Brain Atlas (HBA) is an initiative from Schira et al. (2023) with the goal of creating an *in vivo* atlas of the human brain at (0.25 mm)^3^ resolution from 7T MR images. In order to do so, they have reconstructed a (0.25 mm)^3^ T1w MP2RAGE from 11 individual (0.4 mm)^3^ 1Tx T1w MP2RAGE scans from the same subject. This single subject, ultra-high resolution reconstructed MP2RAGE was used for testing GOUHFI. More details about the initiative and the data can be found online.^‡‡^

Each study was approved by the local review boards of each site/institution and participants of the individual studies signed a written informed consent form before scanning. Complete ethic statements are available at each respective study web pages and publications.

### 2.2 Data processing

#### 2.2.1 Original label map production

All T1w images used in this study were segmented using FastSurferVINN (Henschel et al., 2022) (v2.3.0) with the *–seg_only* flag in order to produce automatic whole brain segmentations into 35 structures/labels. The list of labels produced by FastSurferVINN and used in this work, which follows the standard FreeSurfer lookup table convention (Fischl et al., 2002), is available in Appendix A.1.

Since the T1w images from the SCAIFIELD and UltraCortex dataset have been acquired at UHF-MRI and were used for training in this study, extensive visual quality assurance (QA) has been conducted on all label maps produced by FastSurferVINN. For SCAIFIELD, the pTx MPRAGE images were N4-corrected (Tustison et al., 2010) before being segmented. For both datasets, subjects where low segmentation quality due to motion or important signal inhomogeneities was detected were excluded from the training dataset. For UltraCortex, only 15 of the 78 subjects with (0.8 mm)^3^ MP2RAGE images were assessed good enough to be used for training.

#### 2.2.2 Creation of new label maps and skull-stripped images

Skull stripping is a frequent preprocessing step done in most neuroimaging studies. However, non-brain tissues can remain since the skull-stripping quality is highly dependent on many factors such as MR scanners, sequences used, and image quality (Kleesiek et al., 2016; Pei et al., 2022). In order to simulate a low-quality skull-stripping procedure, new label maps with an “extra-cerebral” label were created from the original label maps produced by FastSurferVINN. First, morphological operations were applied on the brain mask generated by FastSurferVINN. Binary closing was applied to remove possible holes in the mask and dilation was then applied on the filled mask. The number of voxels used for dilation was four (UltraCortex and ABIDE-II) or five (HCP and SCAIFIELD) depending on the resolution of the label maps. These two steps were executed to correct in case of too stringent brain masking. This new mask was then used together with the original mask to create the new extra-cerebral label by assigning all voxels mutually exclusive to the new mask as this new extra-cerebral label (i.e., voxels present in the new mask but not in the original one). Then, the new label map was created by assigning this new label value to the corresponding voxel position in its original label map.

Once the new mask was created, the corresponding input T1w image from which the label map was created was masked to create a skull-stripped version. For multi-contrast datasets such as the HCP and SCAIFIELD, all contrasts were coregistered before applying the masking. The rationale of using skull-stripped training data was based on (1) the fact that some of the datasets were directly shared as skull-stripped data, (2) the inaccessibility to the whole-head segmentation algorithm used in SynthSeg, and (3) the lack of signal outside the brain for UHF-MRI data, making intensity-based whole-head segmentation substantially harder than at 3T.

### 2.3 Generation of synthetic training images

As described in Section 1, the core concept behind SynthSeg is the creation of a training dataset composed solely of synthetic images randomly generated from label maps, regardless of whether they were generated automatically or manually (Billot et al., 2023). In order to create synthetic images, the generative model uses fully randomized parameters from predefined priors, generating images with random (and unrealistic) contrasts, morphologies, artifacts, and noise levels. For an exhaustive explanation behind the generative model developed in SynthSeg, we recommend the reader to consult Billot et al. (2023), since the focus of this section is toward variations from the original model.

**Fig. 1.**
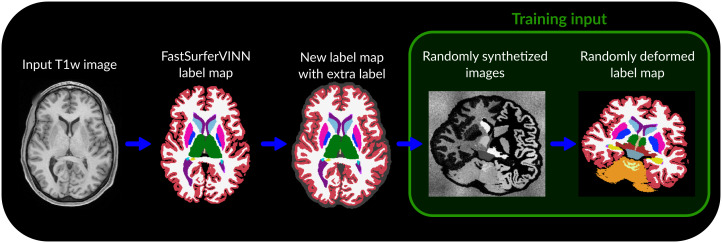
Pipeline used to create the training data for GOUHFI. To produce the training data, the sub-millimeter T1w image was used as input to *FastSurferVINN* in order to create a label map of the whole brain with 35 labels. A new label map was then created by modifying the *FastSurferVINN* output by adding an extra-cerebral label based on the morphologically modified brain mask (dark gray area surrounding the cerebral cortex on the third sub-figure). This new label map was then used as input to the modified generative model from *SynthSeg* to create the randomly deformed version of it. Then, the augmented label map was used to generate the synthetic image where, as explained in Billot et al. (2023), a mean and standard deviation are randomly sampled from a normal distribution to generate a noisy signal for each label iteratively. Ultimately, the extra-cerebral label was kept for generating the synthetic images but excluded from the final label map in order to simulate signal surrounding the cortex such as CSF or remaining non-brain tissues from low-quality skull stripping. Finally, the generated synthetic image with its corresponding deformed label map (from which it was generated) was used as the training data (green box).

In this work, the generative model used in SynthSeg was adapted for UHF images. More precisely, the parameter simulating bias field in the synthetic images was increased from 0.6 to 0.9 to simulate the large signal inhomogeneities frequently observed at UHF, and all parameters related to the randomized downsampling of the synthetic images were disabled to allow the creation of synthetic images at native sub-millimeter resolution. Unlike the original generative model implemented in SynthSeg, in which these structures were excluded, the choroid plexus (both hemispheres), cerebrospinal fluid (CSF), and WM hypointensities labels were incorporated into the generation of synthetic images. The extra-cerebral label was also included in the generative model to synthesize the images, but was excluded from the final label maps. The rest of the generative model was kept as originally proposed and one synthetic image was generated per training label map. An example case is shown in Figure 1, and the parameters of the generative model used in this study are provided in Appendix A.2.

### 2.4 Deep Learning model

#### 2.4.1 Training data

The training dataset used was composed of 206 different subjects from the HCP (n = 80), SCAIFIELD (n = 31), UltraCortex (n = 15), and ABIDE-II (n = 80) datasets with 80% of the subjects randomly assigned as training with the remaining 20% as validation using 5-fold cross-validation. One aspect to achieve contrast agnosticity is the use of real MR images of different contrasts for the validation set, while the synthetic images are used for training only. In other words, if one subject was assigned to the validation set, the input T1w image and corresponding label map were used and not the synthetic image produced by the generative model. Consequently, for the UltraCortex and ABIDE-II datasets, the T1w images with their corresponding label maps were used for validation, whereas for the HCP and SCAIFIELD, the T2w and PDw images were used, respectively, (both coregistered to their corresponding T1w image used for the label map creation).

#### 2.4.2 Network architecture

In this work, the nnUNet framework (v2.4.1) with residual encoder (Isensee et al., 2021, 2024) was used to implement the DL model. The “3D U-Net full image resolution” with “Large Presets” was selected as the configuration, considering the isotropic and sub-millimeter nature of the training data and hardware limitations. More precisely, a 3D U-Net composed of 6 layers with 32, 64, 128, 256, 320, and 320 features, using Leaky ReLU (Maas et al., 2013), 3 × 3 × 3 kernel size for convolutions, with a patch size of 192 × 192 × 160 and a batch size of 2 was used. The loss function was the sum of the Dice and cross-entropy losses (Drozdzal et al., 2016).

The data augmentation step performed by default by the nnUNet was disabled, since extensive data augmentation was already applied by the DR approach used in the generation step of the synthetic images. Since the training dataset was composed of several resolutions, the median resolution of the training dataset ((0.7 mm)^3^) was used as the training resolution for the network. Consequently, images and label maps at different resolutions were externally up- or down-sampled to the median resolution before being fed to the network. For images, 3D cubic spline interpolation was used, whereas 3D linear interpolation with one-hot encoding was used for label maps.

The complete description of all steps performed by the nnUNet framework can be found in Isensee et al. (2021).

#### 2.4.3 Training setup

The model was trained for a total of 500 epochs for each fold, where 1 epoch was defined as 250 random mini-batches fed to the network. The AdamW optimizer (Loshchilov, 2017) was utilized with a base learning rate (LR) of 3 × 10^−4^, decaying following the poly LR scheduler. The total training time was 6 days (1.2 days/fold) using an NVIDIA Ampere A40 GPU with 48 Gb of VRAM.

#### 2.4.4 Inference

Since 5-fold cross-validation was used (i.e., five separate models were trained), an ensembling strategy, where the softmax outputs (i.e., probability for each label) from all models are averaged together, was used to produce a single output label map at inference. Moreover, after training all five models, the default post-processing step proposed by nnUNet of keeping only the largest component for each label was tested with the validation dataset. For all labels, the average Dice score was computed with only the largest component compared with the value with all components for that label. Then, if the average Dice score was improved, the post-processing step would be executed for this label on any data to be inferred. For GOUHFI, this step was applied to all 35 labels except the Left- and Right-Inferior-Lateral-Ventricles.

Since GOUHFI was trained with segmentations produced from FastSurferVINN, and U-Nets are sensitive to spatial localization, all data needed to be reoriented to the Left-Inferior-Anterior (LIA) orientation before being inferred. Moreover, the data to be inferred needed to be resampled to the training resolution of (0.7 mm)^3^ before being processed by the network. The data were then resampled back to native resolution after being segmented. Inference, up/downsampling (if using a different resolution from the one used for training) and post-processing took approximately 60 seconds per 3D volume/image. All these steps are implemented in GOUHFI and all results shown in this work were computed with GOUHFI version 1.1.0 available at https://github.com/mafortin/GOUHFI/releases/tag/1.1.0.

### 2.5 Evaluation metrics

#### 2.5.1 Quantitative evaluation

In order to assess the quality of the segmentations produced by GOUHFI, the Dice-Sørensen Similarity Coefficient (DSC) (Dice, 1945; Sorensen, 1948), which measures the overlap between two segments (with a value of 1 being a perfect overlap between the two segments), was computed with the following equation:

$$DSC=\frac{2\times \left|G\cup P\right|}{G+P},$$ (1)

where G is the ground truth segment, and P is the predicted segment to be compared.

Moreover, the Average Surface Distance (ASD) (Reinke et al., 2024), where a value of 0 represents a perfect alignment of both surfaces evaluated, was computed with the following equation:

$$ASD=\frac{\sum_{i=1}^{N_{G}}d_{G\to P,i}+\sum_{i=1}^{N_{P}}d_{P\to G,i}}{N_{G}+N_{P}}.$$ (2)

Herein, $d_{G\to P,i}$ is the distance from point i on the surface of the ground truth segment to its nearest point on the surface of the predicted segment; $d_{P\to G,i}$ is the distance from point i on the surface of the predicted segment to its nearest point on the surface of the ground truth segment; $N_{G}$ and $N_{P}$ are the total number of points on the ground truth and predicted surfaces, respectively.

Except for the UltraCortex dataset where manual segments were available and the Glasgow dataset where the “inaccurate ground truth” (iGT) was used, the ground truth segmentations in this work were obtained by running FastSurferVINN on the sub-millimeter T1w images for the HCP-YA and the SCA-T1w test datasets. For SCA-MPM contrasts, SynthSeg was used as the ground truth. Thus, in these specific cases, the ground truth was considered a “silver standard” and a perfect DSC of 1.0 was not necessarily desired, especially in cases where FastSurferVINN was prone to face difficulties (e.g., > 3T or < (0.7 mm)^3^ resolution). Nevertheless, FastSurferVINN has previously shown robustness to resolutions < (0.7 mm)^3^ and pTx UHF-MRI data (Fortin et al., 2025). Both FastSurferVINN and SynthSeg are also the only “out-of-the-box” solutions to quantitatively evaluate GOUHFI against in most cases since CEREBRUM-7T requires retraining outside its training domain for every test dataset, and that producing manual delineations for 35 labels for many subjects was outside the scope of this work. For CEREBRUM-7T, the iGT was created by a combination of FreeSurfer, *AFNI3dSeg* (Cox, 1996), and methods from Fracasso et al. (2016) as described in Svanera et al. (2021).

In addition, while FastSurferVINN produces segmentations at the native input image resolution, SynthSeg solely segments images at 1.0 mm^3^, irrespective of the input resolution of the images. In order to obtain label maps at the same resolution as GOUHFI and FastSurferVINN and allow for quantitative comparisons, the same external up-sampling strategy using one-hot encoded 3D linear interpolation for label maps as done for GOUHFI was implemented in-house for SynthSeg.

For calculating DSC and ASD, the choroid plexus (both hemispheres) and WM-hypointensities were excluded since SynthSeg does not segment these labels. Consequently, the lateral and inferior lateral ventricles (both hemispheres) were also excluded since both regions are directly impacted by the presence of the choroid plexus label. Finally, the CSF was also excluded since SynthSeg defines CSF in a completely different way than FastSurferVINN and GOUHFI. In case a label was missing in a label map (i.e., not segmented), DSC and ASD values were set to 0 and NaN, respectively.

#### 2.5.2 STRAT-PARK: Volumetry analysis

To further assess GOUHFI’s performance, a volumetric analysis was performed with the START-PARK dataset. For both HC and PDP, the median group volume for the putamen, amygdala, and hippocampus, normalized by the total intracranial volume (TIV), was computed based on the segmentations produced by FastSurferVINN, GOUHFI, and SynthSeg. The TIV values were computed with SPM12 (Ashburner, 2012) and the region-of-interest (ROI) volumes were computed by multiplying the voxel volume by the number of voxels for each ROI. These ROIs were selected based on the literature for PD (Geng et al., 2006; Junqué et al., 2005; Pieperhoff et al., 2022). A Mann–Whitney U test (Mann & Whitney, 1947) with Bonferroni-corrected p-values (Bonferroni, 1936) was computed to measure the statistical differences between each group for both segmentation tools.

## 3 Results

### 3.1 HCP-YA: Benchmarking against FastSurferVINN and SynthSeg at 3T

**Fig. 2.**
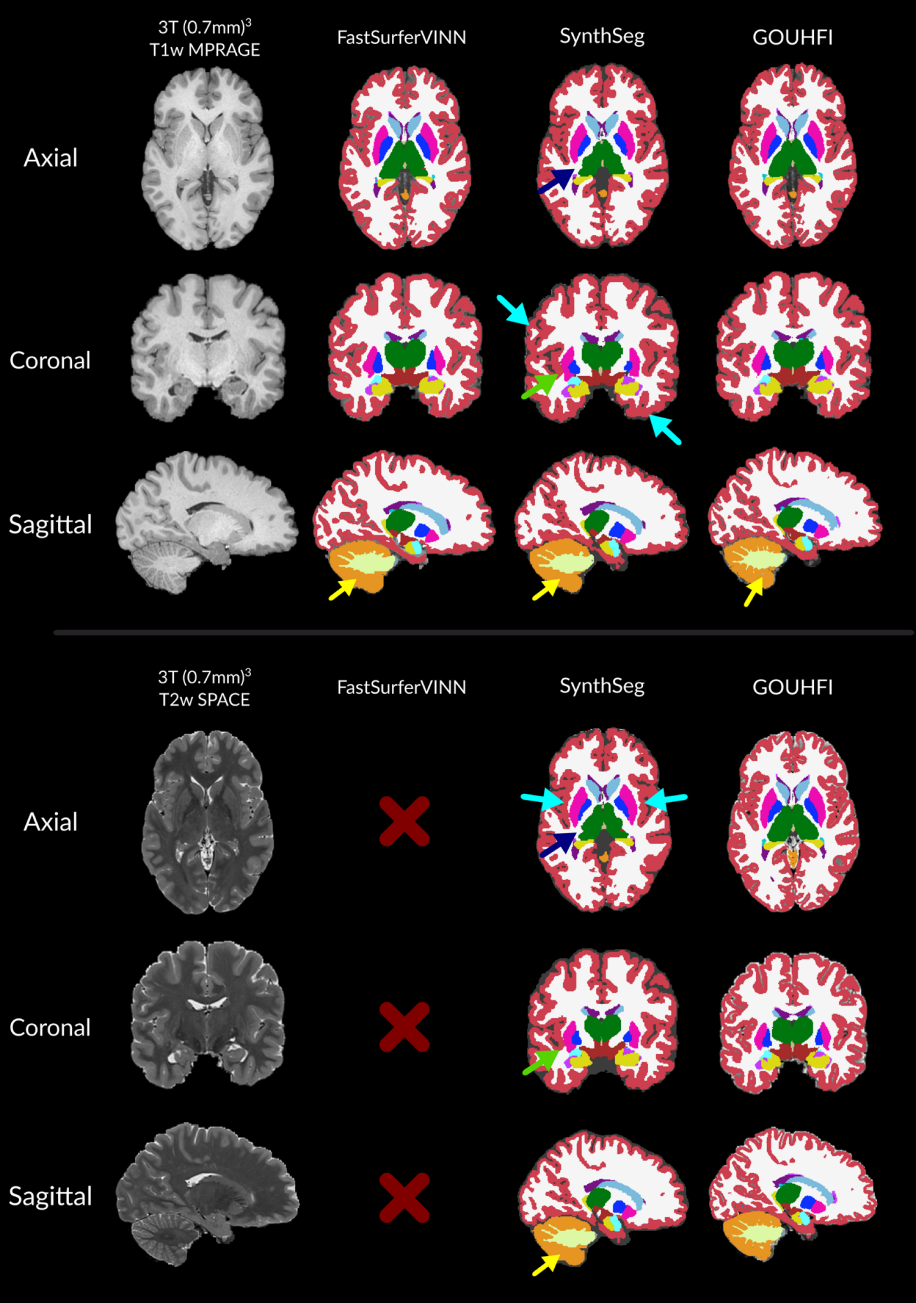
Segmentations produced by *FastSurferVINN* (second column), *SynthSeg* (third column), and GOUHFI (right column) for one subject in all anatomical planes for the T1w (top) and T2w (bottom) contrasts from the HCP dataset (3T). No segmentations are shown for the T2w image for *FastSurferVINN* since it only segments T1w images. Dark blue arrows represent regions of discrepancies for the thalamus region with the ground truth for *SynthSeg*. Turquoise arrows point to cortical regions with limited delineation by *SynthSeg*. Green arrows show systematic errors with *SynthSeg* including the claustrum in the putamen label. Yellow arrows show differences in cerebellum WM segmentation. The labels shown and their colors correspond to the *FreeSurfer* lookup table.

The segmentations produced by SynthSeg and GOUHFI for both T1w and T2w (0.7 mm)^3^ 3T images and by FastSurferVINN for the T1w images only are shown in Figure 2. No segmentation is shown for the T2w images for FastSurferVINN since the technique segments T1w images only. Visually, segmentations produced on both the T1w and T2w images were extremely similar to the ones produced by FastSurferVINN for both SynthSeg and GOUHFI. Visually, differences in cortex and cerebellum WM delineations could be observed for SynthSeg compared with GOUHFI. Moreover, the segmentation boundary of the putamen was extended laterally for SynthSeg compared with both FastSurferVINN and GOUHFI. For SynthSeg, the thalamus delineation was different following a more irregular boundary than the two other techniques as seen with the axial plane. The median and 95% confidence intervals (CI) for the DSC and ASD computed across all subjects (n = 20) and labels with a selected subset are given in Table 2. Overall, GOUHFI produced higher and lower median DSC and ASD values, respectively, for all labels except for the cerebellum WM and cortex compared with SynthSeg.

**Table 2.** Median DSC and ASD values (with 95% CIs) computed for HCP subjects (n = 20) using GOUHFI and *SynthSeg*.

	HCP-T1w		HCP-T2w	
**DSC**	GOUHFI	SynthSeg	GOUHFI	SynthSeg
WM	**0.96** [0.96, 0.97]	0.93 [0.92, 0.93]	**0.95** [0.94, 0.95]	0.90 [0.90, 0.91]
Cortex	**0.92** [0.92, 0.92]	0.88 [0.88, 0.88]	**0.90** [0.90, 0.90]	0.85 [0.85, 0.85]
Putamen	**0.94** [0.93, 0.94]	0.90 [0.89, 0.90]	**0.92** [0.91, 0.92]	0.88 [0.88, 0.89]
Thalamus	**0.93** [0.92, 0.93]	0.91 [0.91, 0.92]	**0.92** [0.91, 0.92]	**0.92** [0.92, 0.92]
Pallidum	**0.85** [0.84, 0.86]	0.82 [0.81, 0.83]	**0.84** [0.83, 0.85]	0.81 [0.79, 0.81]
Cerebellum WM	0.87 [0.87, 0.87]	**0.88** [0.88, 0.89]	0.85 [0.85, 0.85]	**0.87** [0.87, 0.87]
Cerebellum Cortex	0.90 [0.89, 0.90]	**0.93** [0.92, 0.93]	0.89 [0.88, 0.89]	**0.91** [0.90, 0.91]
**Median (27 labels)**	**0.91** [0.90, 0.91]	0.89 [0.88, 0.89]	**0.89** [0.88, 0.89]	0.87 [0.86, 0.87]

	HCP-T1w		HCP-T2w	
**ASD [mm]**	GOUHFI	SynthSeg	GOUHFI	SynthSeg
WM	**0.19** [0.19, 0.19]	0.34 [0.33, 0.34]	**0.27** [0.27, 0.29]	0.41 [0.40, 0.42]
Cortex	**0.26** [0.26, 0.33]	0.36 [0.36, 0.37]	**0.31** [0.31, 0.33]	0.42 [0.42, 0.43]
Putamen	**0.32** [0.32, 0.34]	0.54 [0.53, 0.57]	**0.40** [0.40, 0.43]	0.62 [0.60, 0.66]
Thalamus	**0.52** [0.51, 0.57]	0.56 [0.55, 0.60]	0.61 [0.59, 0.65]	**0.54** [0.54, 0.58]
Pallidum	**0.54** [0.51, 0.58]	0.56 [0.55, 0.61]	**0.58** [0.57, 0.62]	0.62 [0.62, 0.69]
Cerebellum WM	0.67 [0.63, 0.70]	**0.54** [0.51, 0.59]	0.81 [0.79, 0.86]	**0.62** [0.60, 0.67]
Cerebellum Cortex	0.90 [0.87, 0.95]	**0.57** [0.56, 0.59]	0.93 [0.91, 0.99]	**0.66** [0.64, 0.68]
**Median (27 labels)**	**0.45** [0.43, 0.47]	0.50 [0.48, 0.50]	**0.47** [0.51, 0.54]	0.55 [0.54, 0.56]

The ground truth is the segmentation produced by *FastSurferVINN* at native resolution using the T1w images. The highest DSC (and lowest ASD) value is shown in bold.

### 3.2 SCAIFIELD: Contrast- and resolution-agnostic performance at 7T

**Fig. 3.**
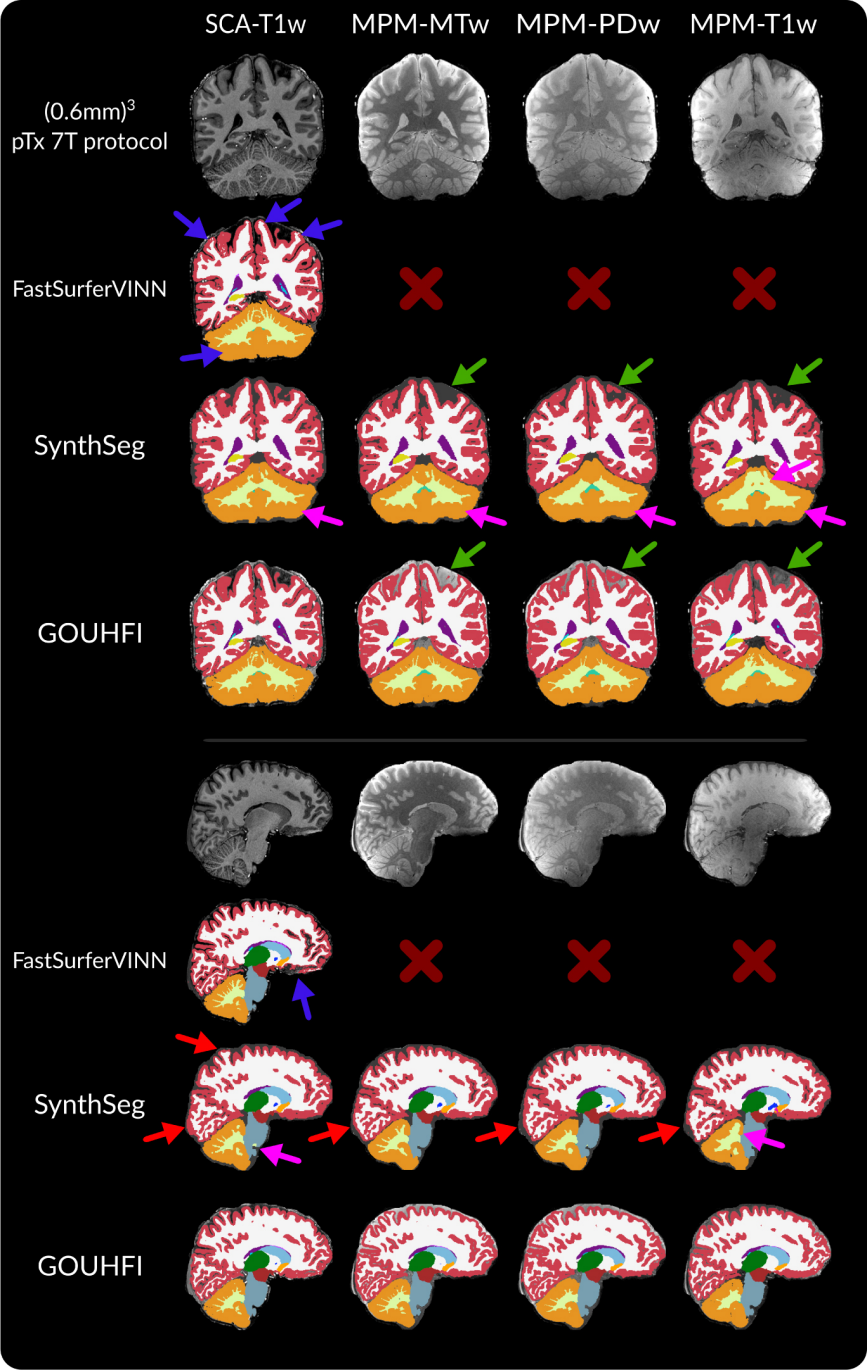
Segmentations produced by *FastSurferVINN* (first row), *SynthSeg* (second row), and GOUHFI (bottom row) for one subject in the coronal (top part) and sagittal (bottom part) planes for the T1w MPRAGE (left), MPM-MTw (second from the left), MPM-PDw (second from the right), and MPM-T1w (right) contrasts from the SCAIFIELD dataset (7T). All images and segmentations have a resolution of (0.6 mm)^3^. The MPRAGE images have been N4-corrected whereas all MPM contrasts have not. Segmentations from *FastSurferVINN* are shown for the T1w image only since it only segments T1w images. Blue arrows represent regions of mislabeling from *FastSurferVINN* (used as ground truth), whereas green arrows show discrepancies between the different MPM contrasts. Pink arrows represent mislabeling of cerebellum WM by *SynthSeg*. Red arrows represent mislabeling between WM and cortex inside the cerebrum where *SynthSeg* overestimated WM segmentation. The labels shown and their colors correspond to the *FreeSurfer* lookup table.

In Figure 3, the segmentations produced by GOUHFI and SynthSeg for all four contrasts and by FastSurferVINN for the T1w MPRAGE are shown. As for the HCP dataset shown in Section 3.1, the segmentations computed by GOUHFI and SynthSeg are visually highly similar to the one from FastSurferVINN for the SCA-T1w. This is also demonstrated quantitatively with the DSC and ASD values reported in the first column of Table 3. Even when using a pTx excitation combined with N4-correction for the MPRAGE images, signs of limited segmentation capacities for 7T images started to appear for FastSurferVINN as shown with the blue arrows in Figure 3. Overall, SynthSeg demonstrated a lower capacity to accurately delineate thin cerebellum WM branches in addition of grossly overestimating size of WM in small folded WM-cortex boundary regions across all contrasts tested compared with GOUHFI (red arrows on Fig. 3). For the SCA-T1w case, GOUHFI had consistently better DSC and ASD values than SynthSeg for all labels tested with a marked difference for the cortex.

**Table 3.** Median DSC and ASD values (with 95% CIs) computed for the four contrasts in the SCAIFIELD dataset (n = 10) using GOUHFI and *SynthSeg*.

	SCA-T1w (GT_FSV_)		MPM (GT_SynthSeg_)		
**DSC**	GOUHFI	SynthSeg	MPM-MTw*	MPM-T1w	MPM-PDw
WM	**0.97** [0.97, 0.97]	0.92 [0.92, 0.92]	0.92 [0.92, 0.93]	0.90 [0.90, 0.90]	0.90 [0.89, 0.90]
Cortex	**0.91** [0.90, 0.92]	0.82 [0.81, 0.82]	0.87 [0.87, 0.88]	0.87 [0.86, 0.87]	0.85 [0.84, 0.86]
Putamen	**0.94** [0.93, 0.94]	0.91 [0.90, 0.91]	0.91 [0.91, 0.92]	0.88 [0.87, 0.89]	0.87 [0.85, 0.88]
Thalamus	**0.94** [0.93, 0.94]	0.92 [0.91, 0.92]	0.91 [0.91, 0.93]	0.93 [0.92, 0.93]	0.92 [0.91, 0.92]
Pallidum	**0.88** [0.86, 0.89]	0.86 [0.84, 0.86]	0.85 [0.80, 0.87]	0.80 [0.78, 0.22]	0.78 [0.74, 0.81]
Cerebellum WM	**0.91** [0.90, 0.91]	0.87 [0.87, 0.88]	0.89 [0.88, 0.90]	0.82 [0.81, 0.83]	0.90 [0.89, 0.90]
Cerebellum Cortex	**0.94** [0.93, 0.94]	0.91 [0.90, 0.91]	0.93 [0.92, 0.93]	0.92 [0.91, 0.92]	0.93 [0.93, 0.93]
**Median (27 labels)**	**0.91** [0.90, 0.91]	0.88 [0.87, 0.88]	0.89 [0.87, 0.89]	0.87 [0.85, 0.86]	0.84 [0.82, 0.84]

	SCA-T1w (GT_FSV_)		MPM (GT_SynthSeg_)		
**ASD [mm]**	GOUHFI	SynthSeg	MPM-MTw*	MPM-T1w	MPM-PDw
WM	**0.13** [0.13, 0.14]	0.36 [0.35, 0.36]	0.44 [0.41, 0.46]	0.54 [0.52, 0.57]	0.57 [0.54, 0.61]
Cortex	**0.23** [0.22, 0.25]	0.45 [0.44, 0.46]	0.50 [0.47, 0.52]	0.52 [0.49, 0.58]	0.61 [0.59, 0.70]
Putamen	**0.26** [0.25, 0.31]	0.47 [0.45, 0.50]	0.41 [0.38, 0.44]	0.54 [0.53, 0.65]	0.63 [0.55, 0.67]
Thalamus	**0.41** [0.39, 0.48]	0.49 [0.49, 0.55]	0.55 [0.51, 0.65]	0.50 [0.48, 0.55]	0.61 [0.57, 0.66]
Pallidum	**0.43** [0.38, 0.46]	0.45 [0.44, 0.51]	0.72 [0.61, 0.87]	0.78 [0.71, 0.82]	0.79 [0.77, 1.01]
Cerebellum WM	**0.32** [0.29, 0.34]	0.45 [0.43, 0.51]	0.51 [0.45, 0.54]	0.69 [0.67, 0.77]	0.53 [0.52, 0.58]
Cerebellum Cortex	**0.49** [0.46, 0.55]	0.65 [0.64, 0.67]	0.59 [0.55, 0.63]	0.65 [0.63, 0.69]	0.65 [0.61, 0.65]
**Median (27 labels)**	**0.34** [0.34, 0.38]	0.48 [0.48, 0.52]	0.50 [0.48, 0.53]	0.56 [0.56, 0.61]	0.64 [0.66, 0.72]

The ground truth for SCA-T1w was the segmentation produced by *FastSurferVINN* at native resolution using the N4-corrected pTx T1w images (GT_FSV_) whereas for the three MPM contrasts, the up-sampled segmentation produced by *SynthSeg* was used (GT_SynthSeg_). For the comparison of GOUHFI and *SynthSeg* versus *FastSurferVINN*, the highest DSC (and lowest ASD) value is shown in bold.

* A subset of four subjects was used for MPM-MTw since several subjects did not include an MTw MPM scan.

**Fig. 4.**
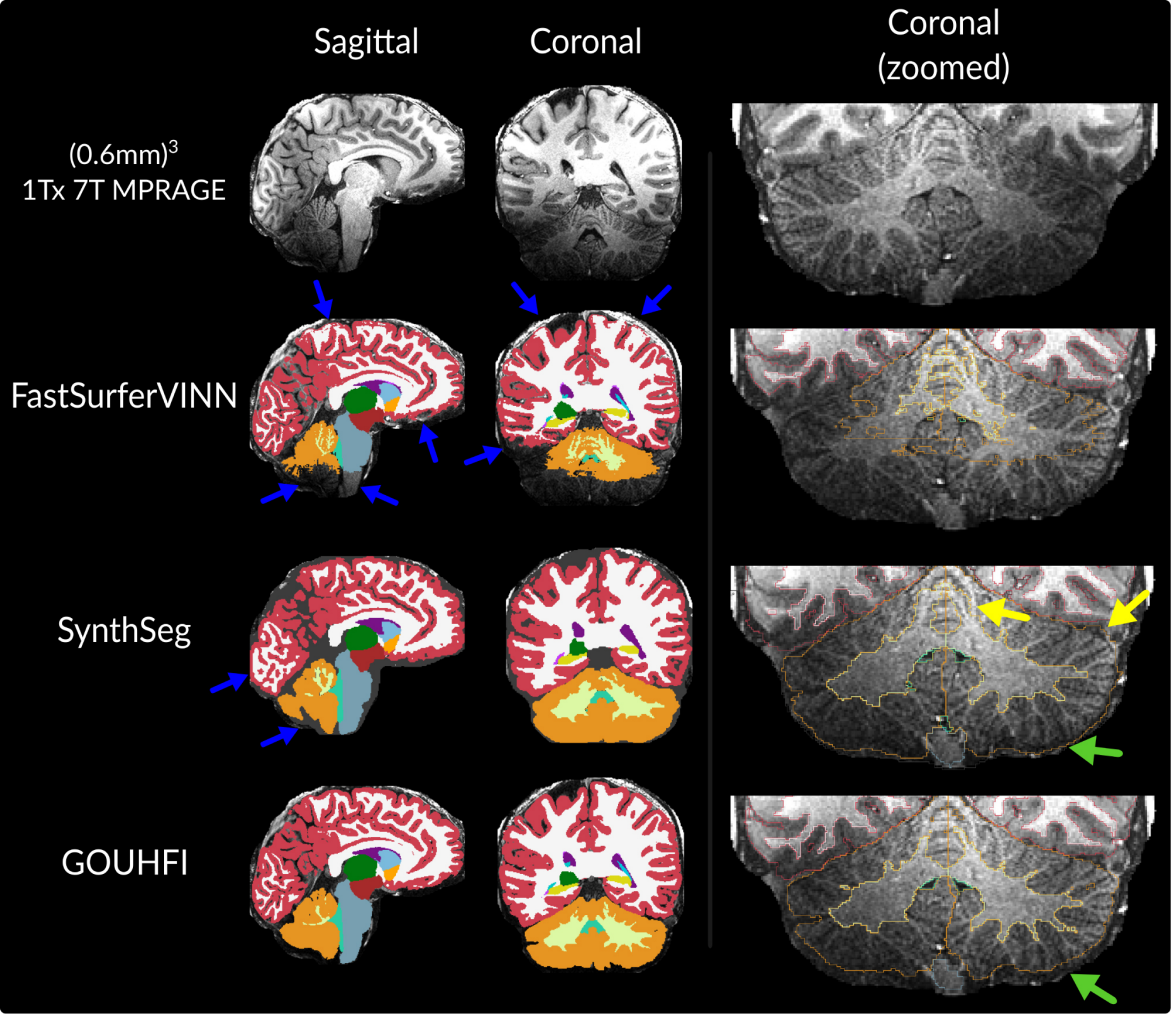
Visual comparison between the segmentations produced by *FastSurferVINN* (second row), *SynthSeg* (third row), and GOUHFI (bottom row) on a 1 Tx MPRAGE acquired for one additional SCAIFIELD subject (7T). Significant signal and contrast inhomogeneities are present. This subject was neither included in the training nor in the testing datasets. The sagittal (first column) and coronal (second column) planes with a zoomed-in version of another coronal slice (third column) with the segmentation borders overlaid are shown. All images and segmentations have a resolution of (0.6 mm)^3^. Blue arrows represent *FastSurferVINN* and *SynthSeg* outputs being affected by signal inhomogeneities. The green arrows show the difference in cerebellar cortex delineation between *SynthSeg* and GOUHFI. Yellow arrows show segmentation errors by *SynthSeg* for the cerebellum WM and cortex. The labels shown and their colors correspond to the *FreeSurfer* lookup table.

The performance of GOUHFI, SynthSeg, and FastSurferVINN on one subject, for which an 1Tx (0.6 mm)^3^ MPRAGE acquisition was acquired (neither part of the training nor the test datasets), is demonstrated in Figure 4. GOUHFI and SynthSeg created substantially better segmentations than FastSurferVINN as expected, especially in regions affected by signal and contrast alterations related to reduced RF transmit inhomogeneities. However, as similarly shown in Figure 3, SynthSeg also showed limited capacity to properly identify the boundary between WM and cortex in some regions compared with GOUHFI.

### 3.3 Glasgow dataset: GOUHFI versus CEREBRUM-7T

**Fig. 5.**
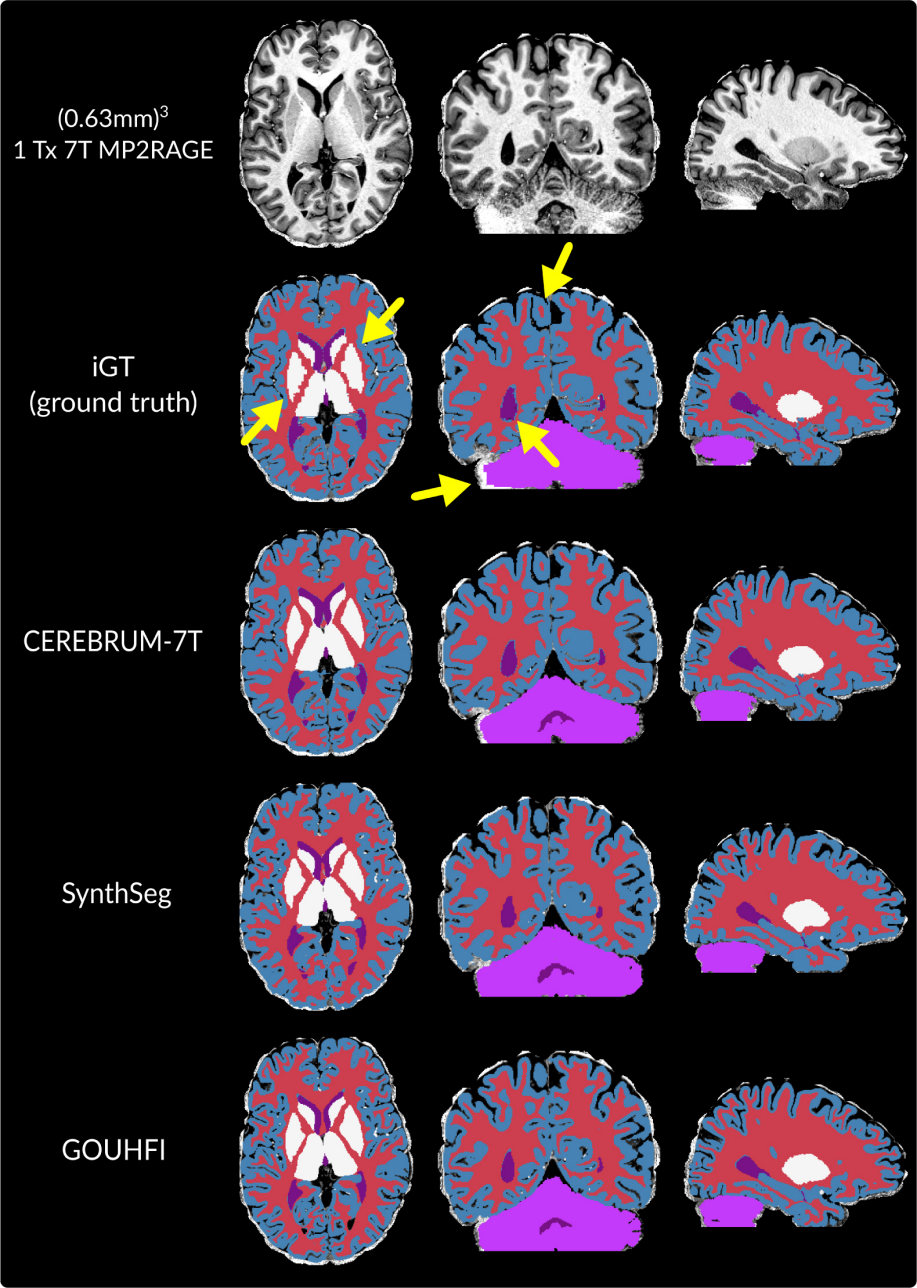
Segmentations produced by *CEREBRUM-7T* (third row), *SynthSeg* (fourth row), GOUHFI (last row) with the corresponding iGT (second row the top) for one subject in all anatomical planes from the test dataset used for *CEREBRUM-7T*. All images and segmentations have a resolution of (0.63 mm)^3^. Yellow arrows point to regions where the iGT (ground truth) seems sub-optimal compared with *CEREBRUM-7T*, *SynthSeg,* and GOUHFI. The labels shown here are gray matter (blue), white matter (red), ventricles (purple), basal ganglia (white), and cerebellum (violet). The brainstem is also segmented but not visible in this figure.

GOUHFI and SynthSeg were tested against CEREBRUM-7T, the only brain segmentation technique optimized for 7T images. The results for one example subject are shown in Figure 5. The DSC and ASD values computed for CEREBRUM-7T, SynthSeg, and GOUHFI against the iGT are reported in Table 4. Both GOUHFI and SynthSeg produced highly similar segmentations between each other and to CEREBRUM-7T, although CEREBRUM-7T being the method with the highest DSC and ASD with the iGT across all labels.

**Table 4.** Median DSC and ASD values (with 95% CIs) for each label across all test cases (n = 21) using segmentations from *CEREBRUM-7T*, GOUHFI, and *SynthSeg*.

**DSC**	CEREBRUM-7T	GOUHFI	SynthSeg
WM	**0.94** [0.94, 0.94]	0.92 [0.91, 0.92]	0.90 [0.89, 0.91]
Cortex	**0.91** [0.90, 0.91]	0.86 [0.86, 0.86]	0.83 [0.83, 0.84]
Basal ganglia	**0.89** [0.89, 0.90]	0.86 [0.86, 0.87]	0.87 [0.86, 0.88]
Ventricles	**0.86** [0.85, 0.87]	0.85 [0.83, 0.86]	0.84 [0.83, 0.86]
Brainstem	**0.93** [0.92, 0.93]	0.91 [0.90, 0.91]	0.91 [0.90, 0.91]
Cerebellum	**0.93** [0.88, 0.94]	0.92 [0.86, 0.92]	0.89 [0.83, 0.90]
**Median**	**0.91** [0.90, 0.91]	0.88 [0.87, 0.89]	0.88 [0.86, 0.87]

**ASD [mm]**	CEREBRUM-7T	GOUHFI	SynthSeg
WM	**0.24** [0.24, 0.25]	0.35 [0.34, 0.36]	0.44 [0.43, 0.45]
Cortex	**0.28** [0.28, 0.29]	0.43 [0.42, 0.44]	0.50 [0.49, 0.50]
Basal ganglia	**0.48** [0.46, 0.50]	0.60 [0.56, 0.63]	0.59 [0.56, 0.63]
Ventricles	**0.35** [0.34, 0.45]	0.39 [0.37, 0.46]	0.38 [0.36, 0.47]
Brainstem	**0.43** [0.41, 0.48]	0.54 [0.51, 0.64]	0.53 [0.50, 0.62]
Cerebellum	**0.68** [0.63, 1.21]	0.90 [0.87, 1.51]	1.26 [1.18, 1.89]
**Median**	**0.38** [0.40, 0.52]	0.49 [0.52, 0.66]	0.51 [0.58, 0.76]

Ground truth is the iGT as described in Svanera et al. (2021). The highest DSC (and lowest ASD) is shown in bold.

### 3.4 MPI-CBS: GOUHFI and SynthSeg performance for ultra-high-resolution and inhomogeneous 7T images

**Fig. 6.**
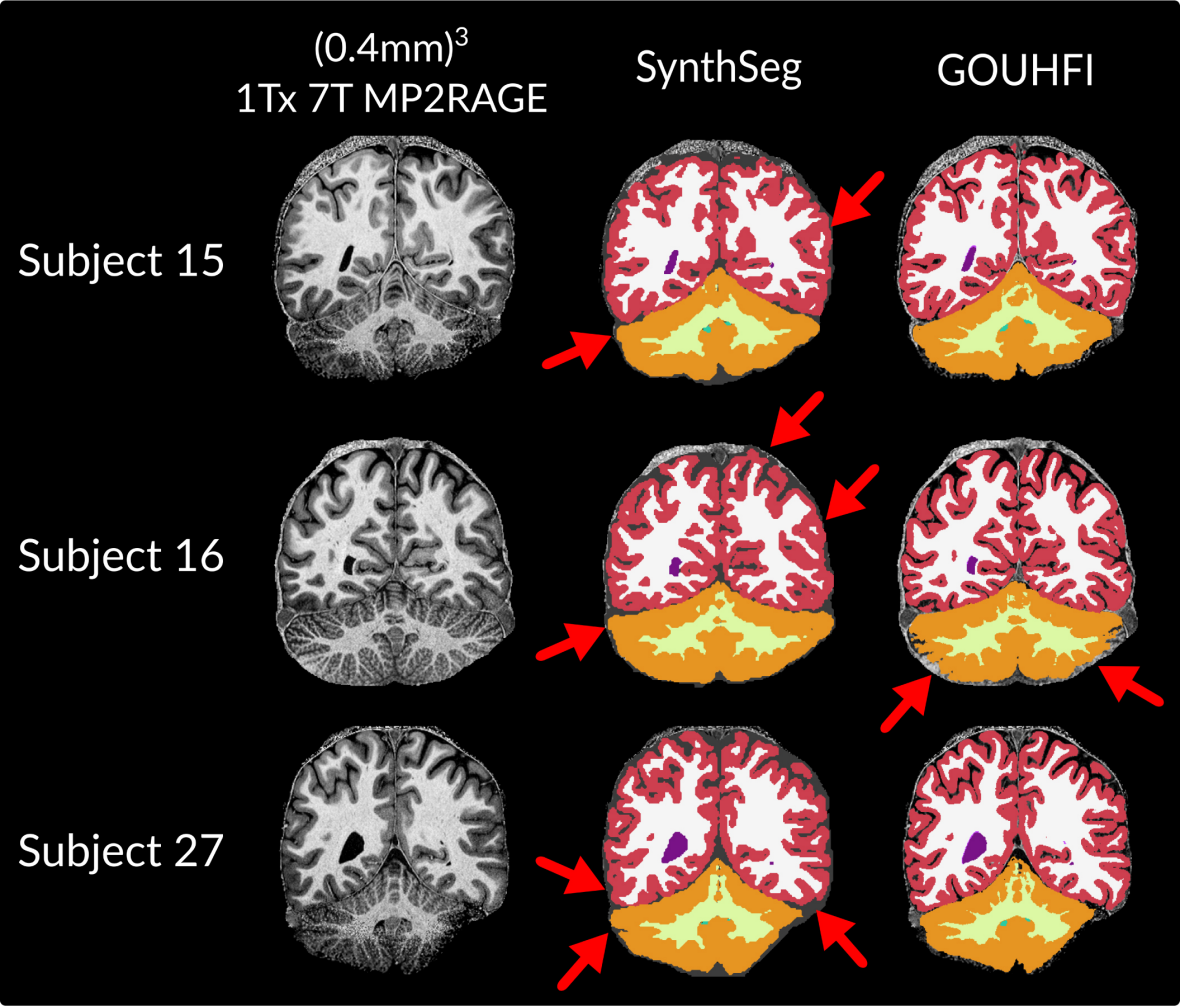
Segmentation results produced by *SynthSeg* (middle column) and GOUHFI (right column) for three subjects from the MPI-CBS dataset in the coronal plane. All images were acquired at 7T with 1Tx (0.4 mm)^3^ MP2RAGE (same resolution for the segmentations). Red arrows point to segmentation errors in cortex and cerebellum cortex delineations.

Three example subjects from the MPI-CBS with (0.4 mm)^3^ 1Tx MP2RAGE acquired at 7T and their corresponding segmentations produced by SynthSeg and GOUHFI are displayed in Figure 6. Although the network segments images at (0.7 mm)^3^ (resolution used by the network for training), the ultra-high resolution of this dataset posed no problem for GOUHFI to properly delineate the brain regions at (0.4 mm)^3^. However, since it was only trained with label maps at 1 mm^3^, SynthSeg showed a limited capacity to show the same level of details especially for the cortex and cerebellum WM branches as also reported in previous sections. Both techniques were able to manage the high level of inhomogeneity and noise present in the images. For subject 16, SynthSeg showed superior identification of the cerebellum cortex in comparison with GOUHFI. However, in all cases, SynthSeg systematically and inaccurately overextended laterally the cerebellum cortex.

### 3.5 UltraCortex: Performance of GOUHFI and SynthSeg against manual white and gray matter delineations at 9.4T

In Table 5, the median DSC and ASD values computed for GOUHFI and SynthSeg against the manual delineations for WM and GM are reported for the UltraCortex dataset. GOUHFI systematically outperformed SynthSeg for every label and sub-dataset, with a substantial advantage for the cortex label with up to 7 Dice points improvement over SynthSeg.

**Table 5.** Median DSC and ASD values (with 95% CIs) computed for the WM and GM segmentations (left and right hemispheres combined) produced by GOUHFI and *SynthSeg* for subjects with manual segments provided in the UltraCortex dataset ((0.6 mm)^3^ MP2RAGE ${\text{n}}_{sub}$ = 8, (0.6 mm)^3^ MPRAGE ${\text{n}}_{sub}$ = 3 and (0.8 mm)^3^ MP2RAGE ${\text{n}}_{sub}$ = 1.).

	(0.6 mm)^3^ MP2RAGE		(0.6 mm)^3^ MPRAGE		(0.8 mm)^3^ MP2RAGE	
**DSC**	GOUHFI	SynthSeg	GOUHFI	SynthSeg	GOUHFI	SynthSeg
White matter	**0.97** [0.97, 0.97]	0.94 [0.94, 0.94]	**0.97** [0.96, 0.97]	0.93 [0.93, 0.94]	**0.95** [-, -]	0.93 [-, -]
Cortex	**0.91** [0.91, 0.91]	0.85 [0.84, 0.85]	**0.90** [0.89, 0.92]	0.85 [0.84, 0.87]	**0.89** [-, -]	0.83 [-, -]

	(0.6 mm)^3^ MP2RAGE		(0.6 mm)^3^ MPRAGE		(0.8 mm)^3^ MP2RAGE	
**ASD [mm]**	GOUHFI	SynthSeg	GOUHFI	SynthSeg	GOUHFI	SynthSeg
White matter	**0.26** [0.25, 0.29]	0.39 [0.37, 0.39]	**0.27** [0.25, 0.32]	0.44 [0.43, 0.44]	**0.35** [-, -]	0.44 [-, -]
Cortex	**0.25** [0.25, 0.27]	0.45 [0.44, 0.46]	**0.29** [0.26, 0.35]	0.44 [0.39, 0.47]	**0.33** [-, -]	0.48 [-, -]

The highest DSC (and lowest ASD) are shown in bold.

### 3.6 STRAT-PARK: Parkinson’s disease volumetry study at 7T

**Fig. 7.**
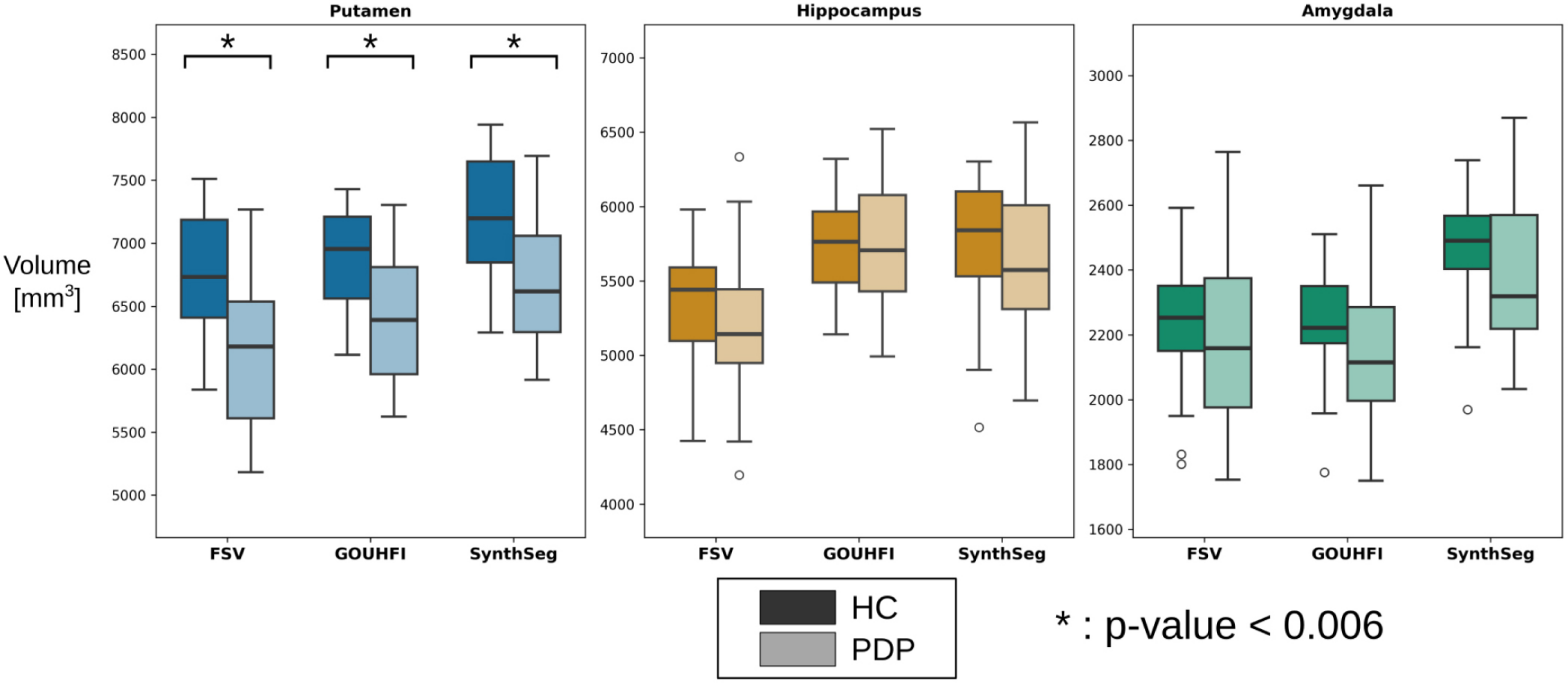
Box plots showing the normalized volumes measured by *FastSurferVINN* (left), GOUHFI (middle), and *SynthSeg* (right) for healthy controls (HC) and Parkinson’s disease patients (PDP) for the putamen, hippocampus, and amygdala. For putamen, the three techniques had a statistically significant difference in volume between HC and PDP after Bonferroni correction.

The volumetric analysis results are shown in Figure 7. The same consistent decrease trend between HC and PDP was observed for all techniques for the putamen, hippocampus, and amygdala. It was only for putamen that all techniques presented a statistically significant difference between both HC and PDP sub-groups. For GOUHFI, the median volumes measured were larger than FastSurferVINN for both HC and PDP, whereas the opposite trend was observed for the amygdala. However, the median volume computed for the amygdala for SynthSeg was considerably and unexpectedly larger than both FastSurferVINN and GOUHFI. The p-values calculated were the following for FastSurferVINN/GOUHFI/SynthSeg: 0.002/0.004/0.0004 for putamen, 0.22/1.0/0.31 for hippocampus, and 0.36/0.06/0.27 for amygdala (Bonferroni-corrected significance threshold: p-value < 0.006).

### 3.7 Human Brain Atlas: Ultra-High Resolution at 7T

**Fig. 8.**
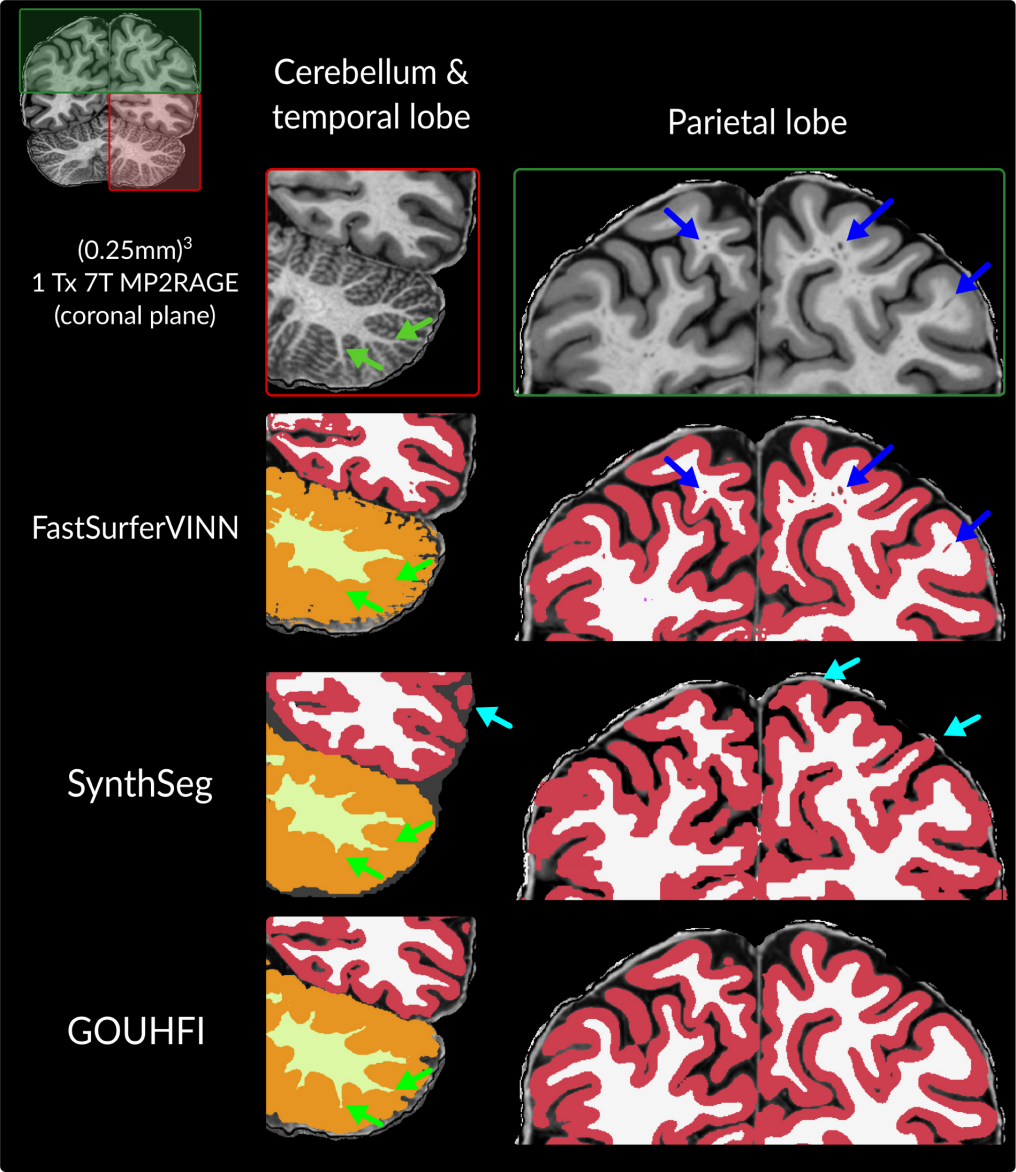
Segmentations produced by *FastSurferVINN* (second row), *SynthSeg* (third row), and GOUHFI (last row) at (0.25 mm)^3^ for the averaged T1w image in the coronal plane for subject 001 from the Human Brain Atlas dataset (7T). The first column shows a zoomed-in version of the cerebellum and temporal lobe, whereas the second column shows the parietal lobe. Green arrows show differences in segmentations between the three methods for fine cerebellar WM branches and their corresponding segmentations, whereas blue arrows show the perivascular spaces inside WM. Turquoise arrows point to cortex segmentation errors for *SynthSeg*.

Zoomed-in coronal views of the (0.25 mm)^3^ segmentations produced by FastSurferVINN, SynthSeg, and GOUHFI for the cerebellum and parietal lobe are presented in Figure 8. For FastSurferVINN, a significant amount of cerebellar WM branches was not segmented even in regions not affected by signal inhomogeneities as shown with the green arrows. Although improvements were noticeable with SynthSeg, the best overall detection and segmentation of cerebellum WM was done by GOUHFI. Moreover, perivascular spaces (PVS) in WM, which become more easily visible at this resolution, were often segmented as background or cortex for FastSurferVINN (blue arrows in Fig. 8), whereas SynthSeg and GOUHFI segmented them as WM. SynthSeg showed limitations in some cortex regions with noticeable mislabeling of non-cortical voxels as cortex. Furthermore, SynthSeg segmentation showed non-smooth, step-like delineations, which FastSurferVINN and GOUHFI did not show.

### 3.8 Impact of label granularity from 3T to UHF-MRI

**Fig. 9.**
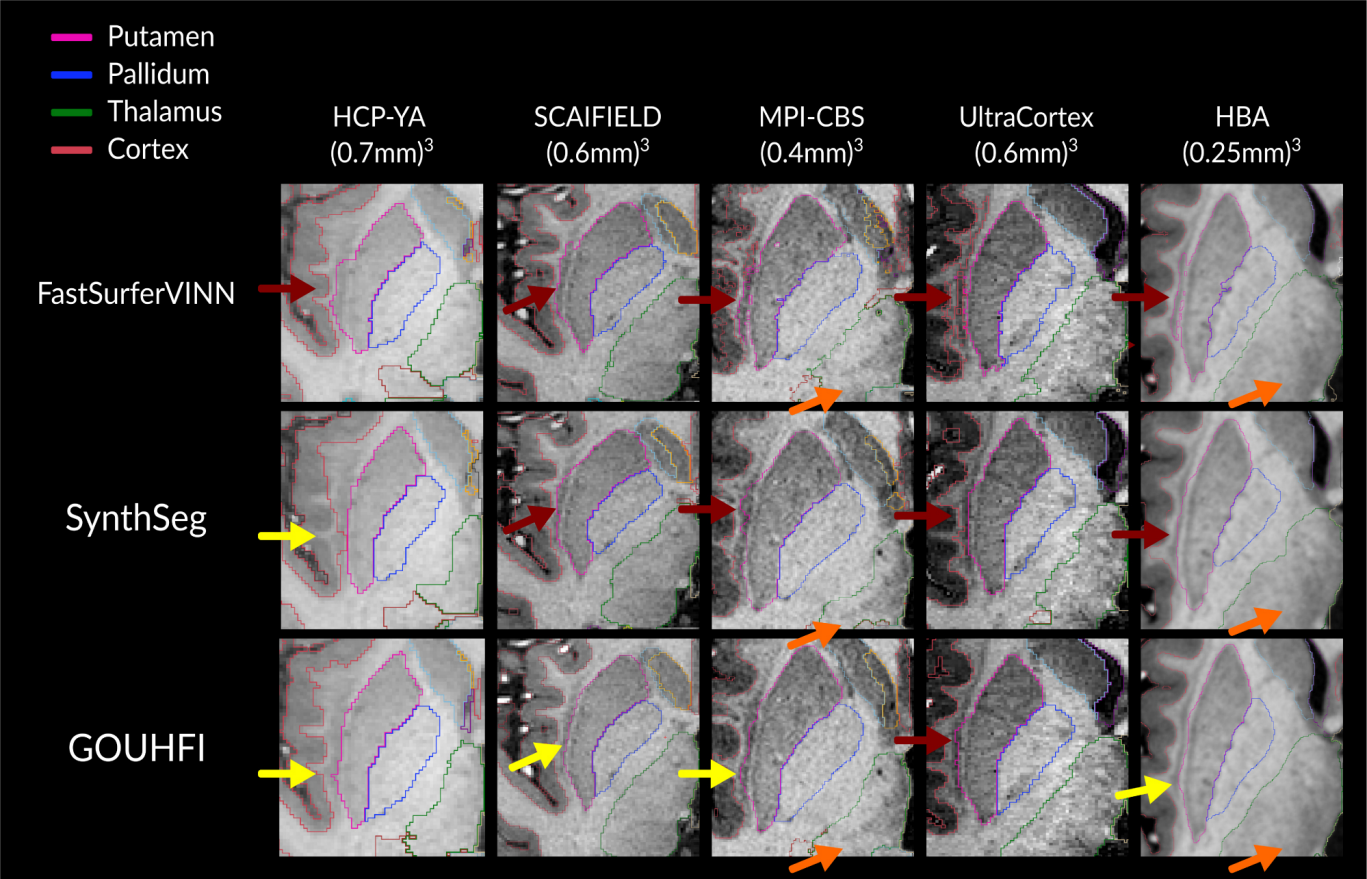
Close-up view of an axial slice showing the segmentations of the putamen, pallidum, thalamus, and cortex in the right hemisphere by *FastSurferVINN* (first row), *SynthSeg* (second row), and GOUHFI (last row) overlaid on the corresponding T1w image used for segmentation. Red arrows show cases where significant parts of the claustrum are segmented as putamen. Yellow arrows show cases where a small portion of the claustrum is included or that the boundary of the putamen is slightly misaligned with its actual border while not including the claustrum. Orange arrows represent ultra-high-resolution cases where subfields of the thalamus can be observed while not being properly segmented.

Figure 9 illustrates the impact of training GOUHFI with label maps generated by FastSurferVINN, tailored to the granularity level typical at 1.5–3T, when applied to UHF images. FastSurferVINN performed the poorest among the three techniques at properly delineating the putamen by systematically including the claustrum in all examples shown. In contrast, although some small portions of the claustrum were still included or, alternatively, the boundary of the putamen was slightly misaligned, GOUHFI performed the best and was the least affected technique by this systematic error among the three. Additionally, for the MPI-CBS and HBA cases where different sub-fields boundaries of the thalamus were discernible, all techniques struggled to accurately identify the thalamus boundary, which resulted in all of them creating a fictitious boundary that did not reflect the internal contrast observed.

## 4 Discussion

In this study, a novel DL-based segmentation technique capable of segmenting brain MR images of any contrast and resolution is proposed. As shown, GOUHFI was able to accurately segment MR images acquired at 3T, 7T, and 9.4T with a total of six different contrasts and seven different resolutions. GOUHFI performed well on highly inhomogeneous 1Tx images acquired at 7T and 9.4T where standard tools are prone to failure. Moreover, GOUHFI demonstrated highly similar performance against domain-specific techniques such as FastSurferVINN or CEREBRUM-7T when tested in their respective domains while also consistently outperforming SynthSeg, the only DL-based contrast-agnostic segmentation tool available. Ultimately, when used to assess its ability to detect volumetric changes in Parkinson’s disease, GOUHFI showed similar performance to FastSurferVINN and SynthSeg to monitor volume losses in accordance with the literature.

### 4.1 Training label maps

**Appendix Fig. A.1.**
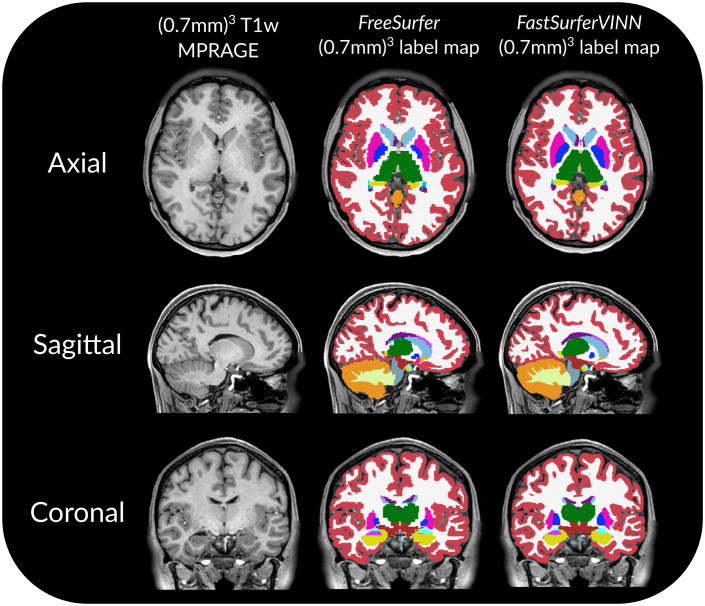
Comparison in the three anatomical planes of the output label maps produced by *FreeSurfer* (middle column) and *FastSurferVINN* (right column) for the same 3T (0.7 mm)^3^ T1w MPRAGE. Despite both label maps being at the same resolution of (0.7 mm)^3^, a difference in delineation quality is easily observable between *FreeSurfer* and *FastSurferVINN*, with the latter producing more refined delineations of anatomical structures. Therefore, *FastSurferVINN* was preferred for the training label maps for GOUHFI.

All previous DL-based segmentation techniques that have been trained on automatically produced label maps have used FreeSurfer to produce the training label maps. To the best of our knowledge, GOUHFI is the first technique to use FastSurferVINN-based label maps in the training dataset. This choice was made based on the fact that the label maps produced by FreeSurfer, even with the sub-millimeter option selected, produced coarse delineations of the labels (see Appendix Fig. A.1 in Appendix A.3). While, in theory, the label maps created by FreeSurfer are at the same resolution as the input images, the “effective resolution” of the label maps is visibly lower. Considering that only sub-millimeter resolutions were used for training and that the intended usage of GOUHFI is for sub-millimeter images, FastSurferVINN was preferred since it produced more refined delineations than FreeSurfer.

In this study, only automatically produced label maps were used for the training corpus, as it was done for FastSurferVINN. While this could be a potential issue when using real UHF images for training, this is not the case when using synthetic images. If minor segmentation errors with respect to the corresponding real T1w input image would be present in the label map (e.g., small parts missing from the cerebellum or temporal lobes due to inhomogeneities), these errors would be “lost” during the creation of the synthetic image for that subject. By design, the synthetic images and corresponding label maps are perfectly aligned with each other. As mentioned in Billot et al. (2023), and again demonstrated in this study, the usage of automatically produced label maps for synthetic training data is not only possible, but highly recommended since it allows to considerably increase the number of training cases. In fact, even for techniques such as FastSurferVINN, which are not based on synthetic training images, the size of the training corpus was shown to be the most important factor to improve the model (Henschel et al., 2022). Ultimately, as mentioned in the Methods section, extensive visual QA was done on the label maps produced by FastSurferVINN for the UHF images before including them in the study (only 15 out of the 78 subjects were kept from the UltraCortex dataset). Since real images were used for validation, we had to make sure that both the quality of the T1w images and label maps were good enough since mismatch between both would negatively impact the validation process.

### 4.2 HCP-YA: Benchmarking against FastSurferVINN and SynthSeg at 3T

For both contrasts tested from the 3T HCP-YA dataset, GOUHFI performed remarkably well and systematically better than SynthSeg. The only ROIs where SynthSeg produced better DSC and ASD values than GOUHFI were for the cerebellar WM and cortex. That can be explained by the similar behavior of SynthSeg to reproduce the limited identification of inferior cerebellar WM branches like FastSurferVINN does. Indeed, GOUHFI detected substantially more cerebellar WM than both FastSurferVINN (reference technique) and SynthSeg, resulting in lower DSC and ASD for both cerebellar WM and cortex. Interestingly, GOUHFI used the same DR approach to create the synthetic training data as SynthSeg. However, for GOUHFI, it resulted in a superior detection of the thin cerebellar WM branches and cortex sulci. Thus, the fact that (1) GOUHFI was trained using only sub-millimeter label maps (with (0.7 mm)^3^ as the training resolution) and (2) the randomized downsampling step as done in SynthSeg was disabled could explain this improved identification of high-resolution anatomy features.

Moreover, the T1w MPRAGE images gave higher DSC and lower ASD values than the T2w images, although only T2w from this dataset was used for the validation set during training. One can argue that T1w was well represented in the validation dataset. However, all other T1w images used for validation were either from a different vendor (ABIDE-II ETH: Philips Achieva with 3D TFE sequence & ABIDE-II EMC: GE MR750 with IR-FSPGR sequence) or different field strength and sequence (UltraCortex: 9.4T with MP2RAGE). Another possible explanation for this could be that it is a consequence of using label maps originally produced from T1w images for the creation of the synthetic training data, which all exhibit the same T1w-visible structures. Ultimately, the finding that GOUHFI produced segmentations with DSC ≥ 0.88 and ASD smaller than 1 voxel over 27 labels and 20 subjects in FastSurferVINN’s domain (i.e., 3T (0.7 mm)^3^ T1w MPRAGE) is a strong indication of its robustness and comparable performance for segmentation tasks even outside GOUHFI’s optimized domain (i.e., UHF-MRI) while also being superior to alternatives such as SynthSeg.

### 4.3 SCAIFIELD: Contrast- and resolution-agnostic performance at 7T

The SCAIFIELD dataset served as an excellent test dataset for GOUHFI considering its variety of contrasts, resolution different from the trained one, and its UHF nature. GOUHFI demonstrated its contrast-agnostic performance by segmenting all four contrasts well. Overall, GOUHFI showed a significantly higher level of details than SynthSeg, especially within the cortex and both the cerebellar WM and cortex labels as similarly reported for the HCP-YA dataset. These observations reinforce the idea that SynthSeg’s use of 1.0 mm^3^ training resolution, combined with the random down-sampling of the training label maps, negatively impacts the quality of the segmentations for UHF images.

For the three MPM contrasts, with SynthSeg as the reference technique, the MPM-PDw image appeared to be the most challenging to segment based on the quantitative metrics computed. However, one could argue that the segmentations displayed in Figure 3 for GOUHFI showed a lower level of detail for the MPM-T1w than the MPM-PDw, especially in the cerebellum. Ultimately, the better quantitative performance of MPM-T1w over MPM-PDw might be explainable by one simple observation: SynthSeg has limited capacity to segment lower contrast regimes at high resolutions such as the MPM-PDw and MPM-T1w, resulting in a poor reference to compare GOUHFI with. Nonetheless, it is important to mention that GOUHFI was also challenged by the low level of contrast in the MPM-T1w, but appeared to segment only visible structures rather than inferring or “hallucinating” invisible regions (cf. pink arrows pointing to cerebellum WM in the coronal view of SynthSeg for the MPM-T1w). Ultimately, the “better” quantitative performance for the MPM-T1w is probably due to a poorer but “matched” performance between SynthSeg and GOUHFI.

Similar to the T2w versus T1w contrast-agnostic comparison using the HCP-YA data, it is interesting to observe that the MPM-PDw dataset showed the lowest quantitative agreement with the reference, while at the same time being the contrast used for the validation dataset for SCAIFIELD data during training.

While being contrast agnostic is a great feature of GOUHFI (and SynthSeg), one inherent aspect of this is its strong generalization to T1w contrast variations. T1w contrast is considered the standard for high-resolution anatomical brain images, however, there is still a wide variety of implementations of T1w contrasts. Indeed, whether it is a difference rising from sequence selection (e.g., MPRAGE vs. MP2RAGE), choice of acquisition parameters (e.g., TI, TE and FA values), or even vendor implementations (e.g., Siemens’ MPRAGE vs. GE IR-FSPGR), T1w can appear quite different across centers or neuroimaging studies. Therefore, as shown in Billot et al. (2023), DL segmentation techniques trained on specific T1w images showed poor generalization to other T1w contrasts, whereas contrast-agnostic techniques such as SynthSeg and GOUHFI performed remarkably well and even better in some cases. Essentially, even if not designed or optimized for T1w contrast, GOUHFI should still be considered as a robust and accurate segmentation option for T1w datasets.

For the SCA-T1w, FastSurferVINN segmentations were still chosen as ground truth over SynthSeg (used for the three MPM contrasts). From ad hoc qualitative assessment of the segmentation quality, FastSurferVINN was deemed a superior segmentation technique over *SynthSeg* even if it was not designed for 7T and < (0.7 mm)^3^ images. Previous work (Fortin et al., 2025) showed that FastSurferVINN performed quite well when N4-correction and pTx pulses were used. Indeed, as shown in Figure 3, the MPRAGE image does not exhibit the typical strong signal inhomogeneities observed at 7T, and the full cerebellum and temporal lobes were properly detected by FastSurferVINN. SynthSeg showed poor boundary detection between WM and cortex in highly gyrified regions (red arrows on sagittal view of Fig. 3) probably due to its low training resolution (+9 Dice points for the cortex label for GOUHFI compared with SynthSeg [0.91 vs. 0.82 respectively]). This was determinant in the decision to not pick SynthSeg as the ground truth for the SCA-T1w case. However, signs of limitations for FastSurferVINN could be observed such as cortical voxels being mislabeled as WM in some cases, or a significant number of cerebellar WM branches not being detected (common issue with SynthSeg). It is also important to mention that the latter issue did not seem to be a 7T-specific issue as cerebellar WM branches appeared to be also difficult to segment at 3T for the T1w images from the HCP dataset (see Fig. 2).

Moreover, it is essential to highlight that even if the theoretical best DSC score achievable is 1, in this work, DSC scores between 0.85 and 0.90 were desired due to the use of a “silver standard” as ground truth. For most test cases tested in this work where FastSurferVINN was set as the reference, it must be highlighted that authors were fully aware that it was not expected to perform well outside the HCP and pTx SCAIFIELD test scenarios. For non-T1w contrasts, SynthSeg was the best and only DL contrast-agnostic technique available to compare GOUHFI with. However, its low training resolution applied to high-resolution images made it a questionable choice as a reference as discussed previously in this section. Thus, in the SCAIFIELD MPM case, the DSC and ASD scores reported against SynthSeg should not be interpreted as direct quantitative assessment of GOUHFI’s performance for UHF-MRI, but rather as a general indicator of how it compared with SynthSeg. Ultimately, this further emphasizes the necessity for novel segmentation techniques to be developed for UHF-MRI.

In all cases tested in this work, GOUHFI demonstrated improved segmentation of cerebellar WM branches over FastSurferVINN and SynthSeg, even in cases such as HCP-T1w where both should be expected to be superior. This makes GOUHFI particularly interesting for neuroimaging studies where the cerebellum is of importance, like for spinocerebellar ataxias (Arruda et al., 2020; Ferreira et al., 2024), whether it is UHF-MRI or not.

Results displayed in Figure 4 showcased GOUHFI’s and SynthSeg’s capacity to segment highly inhomogeneous UHF images, significantly better than FastSurferVINN. Even in the cerebellum region with extremely low signal, GOUHFI was able to properly delineate the cerebellar GM and WM, whereas SynthSeg “over-segmented” the superior region of the cerebellar WM in a similar fashion as for the MPM-T1w as previously discussed. Conversely, even for cortical regions in the parietal and frontal lobes affected by hyperintense signal, GOUHFI accurately detected WM and GM voxels. SynthSeg showed limitations in properly delineating the fine cortical regions with frequent mislabeling resulting in overly segmenting non-cortical voxels as cortex. Overall, SynthSeg showed the same level of resistance to signal inhomogeneity as GOUHFI with most of its limitations probably due to the low training resolution. Since a substantial level of noise was present in the inferior part of the cerebellum, it was not clear where the actual border of the cerebellum was. It is fair to say that SynthSeg proposed a more cautious estimate of it compared with GOUHFI. However, SynthSeg has repetitively shown signs of over-cautiousness with these results (yellow arrows on Fig. 4) and with the MPI-CBS dataset too. Nonetheless, the best identification of the cerebellum GM between SynthSeg and GOUHFI is quite challenging to assess with certainty. Ultimately, the increased random signal bias implemented in the generative model used by GOUHFI for the creation of synthetic training images did not seem to meaningfully modify the overall high inhomogeneity resistance that was already present with SynthSeg’s generative model.

As a result, this resistance of GOUHFI to high levels of noise, granularity, and inhomogeneity is a direct outcome of the use of synthetic images for training. As shown with the example dataset in Figure 1, the synthetic images exhibited similar features to typical UHF images due to the randomly simulated noise and inhomogeneities generated in the images while the corresponding segmentations remained unaffected. This would not be possible if real images were used for training, since the segmentations would be directly affected by the noise and inhomogeneity levels present in the input images.

### 4.4 Glasgow dataset: GOUHFI versus CEREBRUM-7T

Both GOUHFI and SynthSeg performed as well against CEREBRUM-7T and its iGT. Even if the quantitative metrics were slightly lower for GOUHFI and SynthSeg than for CEREBRUM-7T, we would like to argue that this might be the consequence of using the iGT as the ground truth. Indeed, suboptimal delineations were observable in the iGT segments as shown in Figure 5 with the yellow arrows. For instance, coarse delineations were present especially for small cortical regions or the basal ganglia. Moreover, CEREBRUM-7T falsely assigned voxels affected by partial volume effects at the border of ventricles and WM as gray matter, which was also the case for the iGT but not GOUHFI nor SynthSeg. Moreover, the cerebellum segmentation in the iGT was noticeably poor.

Since CEREBRUM-7T is the only segmentation technique optimized for 7T images, one might argue that it should have been the preferred technique for comparison against GOUHFI in this study. However, two main reasons can explain why SynthSeg (or even FastSurferVINN) was preferred. First, CEREBRUM-7T only segments the brain into six labels. For instance, CEREBRUM-7T only generates one label for the basal ganglia. This considerably limits the usability of CEREBRUM-7T in neuroimaging studies where the individual subcortical nuclei such as the thalamus or putamen can be of interest (Rua et al., 2020; Solomon et al., 2017). The same argument applies for the amygdala and hippocampus which were unusually combined with the rest of GM into one single label. Finally, the segmentation of the cerebellum as one label, which does not differentiate between cerebellar WM and GM, is also limiting. At 3T and UHF-MRI, these structures are even frequently segmented into smaller sub-nuclei for more precise analyses (Faber et al., 2022; Haast et al., 2024; Keuken et al., 2013; Morell-Ortega et al., 2024; Plantinga et al., 2018). Considering that CEREBRUM-7T used FreeSurfer v6 to obtain individual subcortical nuclei segments to then recombine them under the basal ganglia as one single segment, it raises the question of why this unusual choice, especially for an UHF-MRI dedicated tool, was made.

The second issue related to CEREBRUM-7T was the technical prerequisites in order to use it. Out-of-the-box, CEREBRUM-7T can only segment images respecting these four requirements: (1) (0.63 mm)^3^ resolution, (2) MP2RAGE sequence, (3) matrix size of 256 × 352 × 224, and (4) images from the Glasgow dataset. Any divergence from one of these four requirements requires fine-tuning (Svanera et al., 2021). For instance, as discussed in Henschel et al. (2022), no de facto standard resolution exists for high-resolution images, and even less at UHF-MRI, making the first requirement quite constraining in a similar fashion as it is for SynthSeg with only 1.0 mm^3^ outputs. While a fine-tuning process requires less time and data than a full DL training, the user still faces practical challenges similar to a full training (i.e., having access to considerable GPU hardware with a significant amount of data curation and preparation) in addition to the prerequisite of a few, already available, high-quality segmentations from their specific dataset. The latter can be interpreted as a circular dependency, where segmentations are actually required in order to produce segmentations. Therefore, in this work, fine-tuning CEREBRUM-7T to every test dataset including 7T data was considered unfeasible due to the complexity of producing high-quality segmentations for these datasets, which exceeded the realistic scope of this work. Moreover, implementing a segmentation technique with these requirements in a clinical setting would be extremely challenging.

Ultimately, considering all practical challenges related to the usage of CEREBRUM-7T on unseen data and its modest number of labels segmented, using CEREBRUM-7T at UHF-MRI is considerably limiting and thus explains its absence for other test datasets in this work.

### 4.5 MPI-CBS: GOUHFI and SynthSeg performance for ultra-high-resolution and inhomogeneous 7T images

Even when sequences such as MP2RAGE are used to reduce the impact of inhomogeneities with 1Tx at 7T, regions such as the cerebellum are frequently affected by poor contrast-to-noise ratios, as shown by Figure 6. Both SynthSeg and GOUHFI performed remarkably well to identify the full cerebellum, with actually a superior identification of the inferior border of the cerebellum by SynthSeg for subject 16. However, SynthSeg systematically and erroneously segmented subarachnoid spaces on both sides of the cerebellum as cerebellum GM for all subjects shown. Additionally, for subject 27, SynthSeg struggled to properly identify and delineate the cortex inside the temporal lobe for both hemispheres.

In the end, this test dataset demonstrated the clear limitation of inferring with a model that was trained with a lower resolution (1.0 mm^3^ or even lower considering the random down-sampling) in terms of properly segmenting fine gyrified cortex regions at ultra-high-resolution like (0.4 mm)^3^. That resulted in frequent mislabeling of the cortex with frequent poor overestimation of the extent of its actual localization (red arrows on Fig. 6).

### 4.6 UltraCortex: Performance of GOUHFI and SynthSeg against manual white and gray matter delineations at 9.4T

Both GOUHFI and SynthSeg showed great accuracy when compared with manual WM and GM segmentations with DSC >0.89 for all three sub-datasets from the UltraCortex 9.4T dataset. However, GOUHFI was consistently superior to SynthSeg across all sub-datasets, with marked superiority for the cortex segmentation with at least six points of improvement on the median DSC. As already discussed in previous sub-sections, this is another example of the limited capacity of SynthSeg to properly segment highly gyrified cortex regions compared with GOUHFI. However, this time, the ground truth is the gold standard with manual delineations, which gives even greater credibility to this observation. After extensive search, this dataset was found to be the only dataset with manual segmentations of complete ROIs available for sub-millimeter images acquired at UHF-MRI. It would have been highly interesting to evaluate GOUHFI against manually segmented subcortical structures at their native sub-millimeter resolution, but we were unable to find such dataset, presumably due to the extensive amount of time and expertise required to execute such a task.

### 4.7 STRAT-PARK: Parkinson’s disease volumetry study at 7T

FastSurferVINN, GOUHFI, and SynthSeg were able to detect volumetric changes between HC and PDP as shown in Figure 7. The consistent decrease in volumes between HC and PDP is in agreement with the literature for these three ROIs (Geng et al., 2006; Junqué et al., 2005; Pieperhoff et al., 2022).

However, one difference observed between FastSurferVINN and both GOUHFI and SynthSeg was the larger median hippocampal volume computed for both HC and PDP compared with FastSurferVINN (middle plot in Fig. 7). Ad hoc qualitative observations of the segmentation results indicated that, in certain subjects with substantially enlarged ventricles, GOUHFI tended to overestimate hippocampal volume by erroneously including portions of the adjacent inferior lateral ventricle inside the hippocampal delineation. That was not observed for FastSurferVINN nor SynthSeg. Nonetheless, this hippocampal over-segmentation did not appear to impact the segmentation quality for the amygdala for GOUHFI. This highlights a potential limitation of the generative model used by GOUHFI, namely its inability to synthesize unhealthy brain anatomies where subtle anatomical deviations from healthy brains can impact its performance. As for any automated segmentation technique, we recommend the users to visually inspect their segmentation results. Incorporating a more diverse training dataset with older subjects could possibly help mitigating this issue. Ultimately, further clinical validation and analyses on more diverse and aged clinical cohorts with different neurological conditions should be done for GOUHFI in the future, but is currently outside the scope of this work.

### 4.8 Human Brain Atlas: Ultra-High Resolution at 7T

Reaching ultra-high-resolution levels like for the (0.25 mm)^3^ MP2RAGE images from the Human Brain Atlas dataset allows for the visualization of PVS. Indeed, PVSs in healthy subjects have diameters between 0.13 mm and 0.96 mm, with the majority being below 0.5 mm (Zong et al., 2016). GOUHFI and SynthSeg did not segment any PVS (it was included in WM), whereas FastSurferVINN segmented most of them and labeled them as background or cortex. Whether PVS should be segmented as part of WM is subject to discussion. However, segmenting them as cortex is incorrect. Especially at UHF-MRI, researchers should start considering specific inclusion of PVS in label maps, in particular, as the number of studies about PVS has drastically increased in the recent years (Feldman et al., 2018; George et al., 2021; Kilsdonk et al., 2015; Wardlaw et al., 2020).

The (0.25 mm)^3^ segmentations produced by GOUHFI showed great delineation of the structures despite the fact that this resolution was considerably outside the training resolution of (0.7 mm)^3^. Conversely, SynthSeg did not show the same level of delineation for the segmentations, even if the same up-sampling approach as GOUHFI was used. These results further demonstrated the clear improvement in delineation quality by using a higher training resolution for GOUHFI over SynthSeg. In Figure 8, one can observe the cortex segmentation created by SynthSeg, even if up-sampled to (0.25 mm)^3^, has jagged contours and several regions of overextending the cortex into the bordering CSF (turquoise arrows). Both these behaviors were not observed for GOUHFI. The substantial difference in voxel volume between the output resolution of SynthSeg and the input HBA image (64 times bigger voxels) made the jagged contours more apparent, although such artifacts were also present, albeit less visibly, at other sub-millimeter resolutions.

Indeed, GOUHFI (and SynthSeg) uses an “*external scaling*” (exSA) approach to deal with any resolution instead of the “*internal scaling*” (or VINN) approach as proposed in FastSurferVINN. While in Henschel et al. (2022) the results were consistently better for the VINN approach over the exSA approach for all datasets shown in their figure 8 (and the corresponding Table 9), none of the datasets showed a significantly better performance statistically, with only minimal improvement of the mean values compared with the standard deviations. In fact, for the subcortical structures of the ADNI dataset, the DSC and ASD values reported in Table 9 were actually higher for exSA over the VINN approach, which is in disagreement with the results reported in their figure 8. Overall, the differences reported between the exSA and VINN approaches were larger for cortical than subcortical structures (i.e., only two labels, left- and right-cortex, compared with the 33 other labels in this work).

One technical challenge of using the VINN technique (like FastSurferVINN) is the substantial increase in required GPU hardware to segment ultra-high resolutions, like the (0.25 mm)^3^ used here, since the full matrix is fed to the network. For instance, the GPU hardware used in this work was not powerful enough to segment the (0.25 mm)^3^ images due to VRAM limitations. This resulted in the required usage of CPU resources to segment the (0.25 mm)^3^ images using FastSurferVINN, which increased computation time by a factor of 78. While this was not an issue for a single subject dataset, using the VINN approach might be limiting for the increased matrix size of images with ultra-high resolutions like the HBA case and is something to consider for larger datasets or researchers with limited access to GPU hardware.

### 4.9 Impact of label granularity from 3T to UHF-MRI

As reported in Valabregue et al. (2024), FreeSurfer and FastSurferVINN have been shown to erroneously include a substantial portion of the claustrum within the putamen segmentation. Since both SynthSeg and GOUHFI were trained using label maps produced by FreeSurfer and FastSurferVINN, respectively, the same pattern should have been expected especially due to the higher contrast at UHF. Surprisingly, as demonstrated in Figure 9, GOUHFI, while not being able to perfectly delineate the putamen, did not exhibit as poor delineations of the putamen as FastSurferVINN, and overall better than SynthSeg. Nevertheless, when tested on ultra-high resolution and high T1w contrast like for the MPI-CBS and UltraCortex datasets, GOUHFI performed suboptimally in a similar fashion as SynthSeg. One additional related issue, specific to SynthSeg, is its poor delineation of the cerebral cortex which frequently resulted in the cortex and putamen being directly segmented next to each other (cf. yellow arrow on the HCP-YA example).

Moreover, an issue arising from using label maps defined at the 3T-granularity level is the absence of sub-field distinction for some subcortical nuclei. Given that UHF-MRI offers increased resolution and contrast, this can become a problem for some structures such as the thalamus, hippocampus, or amygdala. Indeed, especially for the MPI-CBS and HBA cases with ultra-high resolution at 7T, different contrasts were visible and present inside the thalamus label. While this single thalamus label definition was adequate at 1.5 and 3T, this definition becomes limiting in some instances at 7T with ultra-high resolution and contrast as shown here.

Ultimately, the limitations observed for both the claustrum and thalamus in FastSurferVINN, SynthSeg, and GOUHFI underscore the need for label definitions adapted to the granularity of UHF images. This adaptation, not widely implemented in large-scale automatic segmentation tools, will be essential in order to accurately capture the sub-field nature of subcortical nuclei. Promising new tools such as *NextBrain* (Casamitjana et al., 2024) could help implement updated label definitions in the future for automatic UHF segmentation techniques such as GOUHFI.

### 4.10 Limitations

It is important to acknowledge certain limitations of our study, such as the limited availability of reference techniques to compare GOUHFI with. As discussed in Section 4.4, it would have been preferable if CEREBRUM-7T would have offered the same out-of-the-box implementation like FastSurferVINN or SynthSeg (albeit the extra external up-sampling step that had to be added by the authors in order to enable comparisons for SynthSeg). Additionally, it is worth repeating that CEREBRUM-7T produces only 6 labels, whereas GOUHFI produces 35 (following FreeSurfer/FastSurferVINN label convention). This allows for a considerably larger number of regions to use for quantitative analyses with GOUHFI, which is especially of interest at UHF-MRI. Ultimately, this lack of reference segmentation techniques at UHF-MRI further manifests the need for novel techniques to be developed.

A common issue faced by all novel segmentation techniques is the sparsity of real ground truth segmentations to use for testing. In this work, manual segmentations were only available for the UltraCortex dataset and two labels only. For other quantitative analyses, either FastSurferVINN segments computed on T1w images or SynthSeg were used as a “silver standard” or, for CEREBRUM-7T, the iGT was used. To the best of our knowledge, no dataset available online offers 3D sub-millimeter manual segmentations for several subcortical labels at UHF-MRI. Moreover, producing our own manual segmentations would have been extremely time consuming and required expertise outside the scope of this work. In addition, manual segmentations are prone to inter- and intra-expert variability (Deeley et al., 2011).

Despite GOUHFI being able to segment any contrast and resolution tested, input images still need to be skull stripped unlike similar techniques (FastSurferVINN, SynthSeg, or CEREBRUM-7T). The reasons behind this requirement are that, first, some training data were already skull stripped when accessed, and second, segmenting extra-cerebral labels as in Billot et al. (2023) was not easily obtainable for UHF images due to signal inhomogeneities outside the brain. Extra-cerebral labels are required in order to generate synthetic contrasts for the whole head and such tools are not readily available for UHF images. In contrast, considering that skull stripping is a quite common step for neuroimaging pipelines, and that it has been extensively developed and improved recently with the arrival of DL-based techniques, we strongly believe that it should not limit the usability of GOUHFI in practice. Indeed, a wide variety of robust and extensively tested options are freely and easily available online such as BET, HD-BET, SynthStrip, ROBEX, ANTsPyNet, and MONSTR (Hoopes et al., 2022; Iglesias et al., 2011; Isensee et al., 2019; S. Roy et al., 2017; Smith, 2002; Tustison et al., 2021). Nonetheless, possible errors in skull stripping can impact the quality of the segmentation results and we recommend users to assess the skull stripping on their images before using GOUHFI and use a consistent procedure for a given image type.

A potential drawback of GOUHFI, designed for UHF-MRI, is the fact that cortex parcellation is not performed. This can be a limitation for researchers using functional MRI (fMRI) at UHF where its advantages, compared with 3T, have been shown Beisteiner et al. (2011). In addition, as previously mentioned, all training and most of the test data in this study consisted of MR images of young healthy subjects. While the volumetry results on PDP versus HC indicate that GOUHFI is comparable with FastSurferVINN, they also pointed to some potential problems of GOUHFI related to enlarged lateral ventricles. A broad and systematic evaluation of the performance of GOUHFI in the presence of deviating anatomies and various pathologies was outside the scope of the current study, but should be done in a separate follow-up study. Such a study should also consider a retraining of GOUHFI with added data from patient studies in the training corpus. This could, for example, include open-access databases with neurological disorders such as the OASIS or ADNI databases (Jack Jr et al., 2008; Marcus et al., 2007). Originally excluded due to their lower resolutions (i.e., 1 mm^3^), these datasets could offer anatomical variations, like enlarged ventricles, that can be impossible to synthesize with the generative model and, thus, improve the robustness of GOUHFI to a wider range of brain anatomies. Ultimately, addressing the limitations related to the cortex parcellation, lack of anatomical diversity in the training data, and thorough testing of GOUHFI on clinical cohorts with pathologies represents the main focus of future work.

## 5 Conclusions

In summary, we propose GOUHFI, a novel DL-based segmentation technique capable of segmenting MR images acquired with various contrasts, resolutions, and even field strengths. GOUHFI was able to segment all six resolutions and seven contrasts tested in this work. The usage of synthetic images for training enabled the segmentation of images acquired at 3T, 7T, and 9.4T. At 3T, when compared with FastSurferVINN, GOUHFI gave an average DSC of 0.89 for both T1w and T2w images, demonstrating great performance at lower field strengths and superiority over SynthSeg, although developed for UHF applications. At 7T, GOUHFI was able to segment five different contrasts and showed similar performance to CEREBRUM-7T while being substantially more generalizable and practical for the UHF-MRI context. At 9.4T, GOUHFI demonstrated high agreement with manual segmentations with an average DSC of 0.93 over 12 subjects versus 0.89 for SynthSeg. Despite SynthSeg exhibiting decent performance at UHF with high inhomogeneity resistance, SynthSeg lacked the necessary granularity required at UHF in its output segmentations, likely due to the low training resolution. Ultimately, by being trained on synthetic images randomly generated from only sub-millimeter label maps, GOUHFI was able to develop contrast- and resolution-agnostic capabilities adapted to the UHF-MRI reality with, in addition, a significant resistance to noise and signal inhomogeneities, which have been a major challenge for automatic segmentation at UHF-MRI until now. For this initial version of GOUHFI, the training and testing were predominantly conducted using data from healthy subjects. While this will be addressed in its next iteration, it is important to consider this factor when applying GOUHFI to patient cohorts.

## Data Availability

The source code for GOUHFI is available on GitHub at https://github.com/mafortin/GOUHFI. All MRI datasets used in this article are open source repositories freely available through the links provided in footnotes of Section 2 except for the SCAIFIELD and STRAT-PARK datasets. The latter are not publicly available due to data protection regulations.

## Footnotes

*
https://www.humanconnectome.org/storage/app/media/documentation/s1200/HCP_S1200_Release_Reference_Manual.pdf

†
https://www.humanconnectome.org/study/hcp-young-adult

‡
https://www.dzne.de/en/research/projects/scaifield/about/

§
https://www.ultracortex.org/

**
http://fcon_1000.projects.nitrc.org/indi/abide/abide_II.html

††
https://search.kg.ebrains.eu/instances/Dataset/2b24466d-f1cd-4b66-afa8-d70a6755ebea

‡‡
https://osf.io/ckh5t/

## Appendix A.1. List of the Structures and Corresponding Label Values Segmented by Gouhfi

**Appendix Table A.1.** Label name and index of all structures segmented by GOUHFI. Lh and rh stands for Left- and Right-Hemisphere, respectively.

Segmented structure	Label index
Cerebral white matter (lh)	1
Cerebral cortex (lh)	2
Lateral ventricle (lh)	3
Inferior lateral ventricle (lh)	4
Cerebellar white matter (lh)	5
Cerebellar cortex (lh)	6
Thalamus (lh)	7
Caudate (lh)	8
Putamen (lh)	9
Pallidum (lh)	10
3rd-ventricle	11
4th-ventricle	12
Brain stem	13
Hippocampus (lh)	14
Amygdala (lh)	15
CSF	16
Accumbens (lh)	17
Ventral DC (lh)	18
Choroid plexus (lh)	19
Cerebral white matter (rh)	20
Cerebral cortex (rh)	21
Lateral ventricle (rh)	22
Inferior lateral ventricle (rh)	23
Cerebellar white matter (rh)	24
Cerebellar cortex (rh)	25
Thalamus (rh)	26
Caudate (rh)	27
Putamen (rh)	28
Pallidum (rh)	29
Hippocampus (rh)	30
Amygdala (rh)	31
Accumbens (rh)	32
Ventral DC (rh)	33
Choroid plexus (rh)	34
WM-hypointensities	35
Extra-cerebral	36*

* While required to generate the synthetic images used for training, the extra-cerebral label is not segmented by GOUHFI.

## Appendix A.2. Parameters of the Generative Model for Synthetic Image Creation

**Appendix Table A.2.** Values of the parameters used in this study for the generative model.

Parameter	Value
${\text{a}}_{rot}$	-20
${\text{b}}_{rot}$	20
${\text{a}}_{sc}$	0.8
${\text{b}}_{sc}$	1.2
${\text{a}}_{sh}$	-0.015
${\text{b}}_{sh}$	0.015
${\text{a}}_{tr}$	-30
${\text{b}}_{tr}$	30
${\text{b}}_{nonlin}$	4.0
${\text{a}}_{\mu }$	0
${\text{b}}_{\mu }$	255
${\text{a}}_{\sigma }$	0
${\text{b}}_{\sigma }$	35
${\text{b}}_{B}$	0.9
$\sigma_{\gamma }^{2}$	0.4
${\text{r}}_{HR}$	None
${\text{b}}_{res}$	None
${\text{a}}_{\alpha }$	None
${\text{b}}_{\alpha }$	None

Intensity parameters assume inputs in the [0, 255] interval, rotations are expressed in degrees with spatial measures in millimeters. More details about these parameters are provided in Billot et al. (2023).
